# Functional Polarity of Microvascular Brain Endothelial Cells Supported by Neurovascular Unit Computational Model of Large Neutral Amino Acid Homeostasis

**DOI:** 10.3389/fphys.2018.00171

**Published:** 2018-03-13

**Authors:** Mehdi Taslimifar, Stefano Buoso, Francois Verrey, Vartan Kurtcuoglu

**Affiliations:** ^1^The Interface Group, Institute of Physiology, University of Zurich, Zurich, Switzerland; ^2^Epithelial Transport Group, Institute of Physiology, University of Zurich, Zurich, Switzerland; ^3^Institute for Diagnostic and Interventional Radiology, Zurich University Hospital, Zurich, Switzerland; ^4^Zurich Center for Integrative Human Physiology, University of Zurich, Zurich, Switzerland; ^5^National Center of Competence in Research, Kidney.CH, Zurich, Switzerland; ^6^Neuroscience Center Zurich, University of Zurich, Zurich, Switzerland

**Keywords:** blood brain barrier, neurovascular unit, amino acid transporter, large neutral amino acid, SLC7A5/LAT1

## Abstract

The homeostatic regulation of large neutral amino acid (LNAA) concentration in the brain interstitial fluid (ISF) is essential for proper brain function. LNAA passage into the brain is primarily mediated by the complex and dynamic interactions between various solute carrier (SLC) transporters expressed in the neurovascular unit (NVU), among which SLC7A5/LAT1 is considered to be the major contributor in microvascular brain endothelial cells (MBEC). The LAT1-mediated trans-endothelial transport of LNAAs, however, could not be characterized precisely by available *in vitro* and *in vivo* standard methods so far. To circumvent these limitations, we have incorporated published *in vivo* data of rat brain into a robust computational model of NVU-LNAA homeostasis, allowing us to evaluate hypotheses concerning LAT1-mediated trans-endothelial transport of LNAAs across the blood brain barrier (BBB). We show that accounting for functional polarity of MBECs with either asymmetric LAT1 distribution between membranes and/or intrinsic LAT1 asymmetry with low intraendothelial binding affinity is required to reproduce the experimentally measured brain ISF response to intraperitoneal (IP) L-tyrosine and L-phenylalanine injection. On the basis of these findings, we have also investigated the effect of IP administrated L-tyrosine and L-phenylalanine on the dynamics of LNAAs in MBECs, astrocytes and neurons. Finally, the computational model was shown to explain the trans-stimulation of LNAA uptake across the BBB observed upon ISF perfusion with a competitive LAT1 inhibitor.

## Introduction

The blood-brain barrier (BBB) is a truly dynamic interface separating the brain from the bloodstream. It is formed by highly specialized microvascular brain endothelial cells (MBECs) connected by tight junctions forming brain capillaries. The BBB endothelium together with the astrocytes and neurons are the fundamental elements of the neurovascular unit (NVU) system.

Numerous solutes move across the NVU cell membranes with various transport mechanisms. While small lipophilic molecules can diffuse, larger and hydrophilic solutes, such as amino acids (AAs), need the assistance of specialized carrier proteins to cross the membrane, for instance amino acid transporters (AATs) (Abbott et al., 2006). NVU-AATs are expressed at both luminal and abluminal membranes of the MBECs, as well as on astrocytes and neurons. The NVU-AATs mediate the transfer of particular amino acids with different transport mechanisms: antiporters, for example, exchange some AAs for others across the membrane, while symporters cotransport AAs together with ions along the ions' electrochemical gradient (Taslimifar et al., 2017). Taken together, different classes of NVU-AATs constitute an integrated dynamic system controlling the homeostasis of AAs such as large neutral amino acids (LNAAs: L-tyrosine, L-leucine, L-isoleucine, L-phenylalanine, L-histidine, L-valine, L-tryptophan, and L-methionine) in the brain interstitial fluid (ISF). The homeostasis maintenance of LNAA concentrations, which have been shown to be asymmetrically distributed in the plasma and individual NVU compartments (Kandera et al., 1968; Currie et al., 1995; Dolgodilina et al., 2015), is of particular importance due to their crucial role in the central nervous system (CNS), for instance as precursors of key neurotransmitters such as Dopamine, Serotonin, and Histamine.

**Figure 1:**
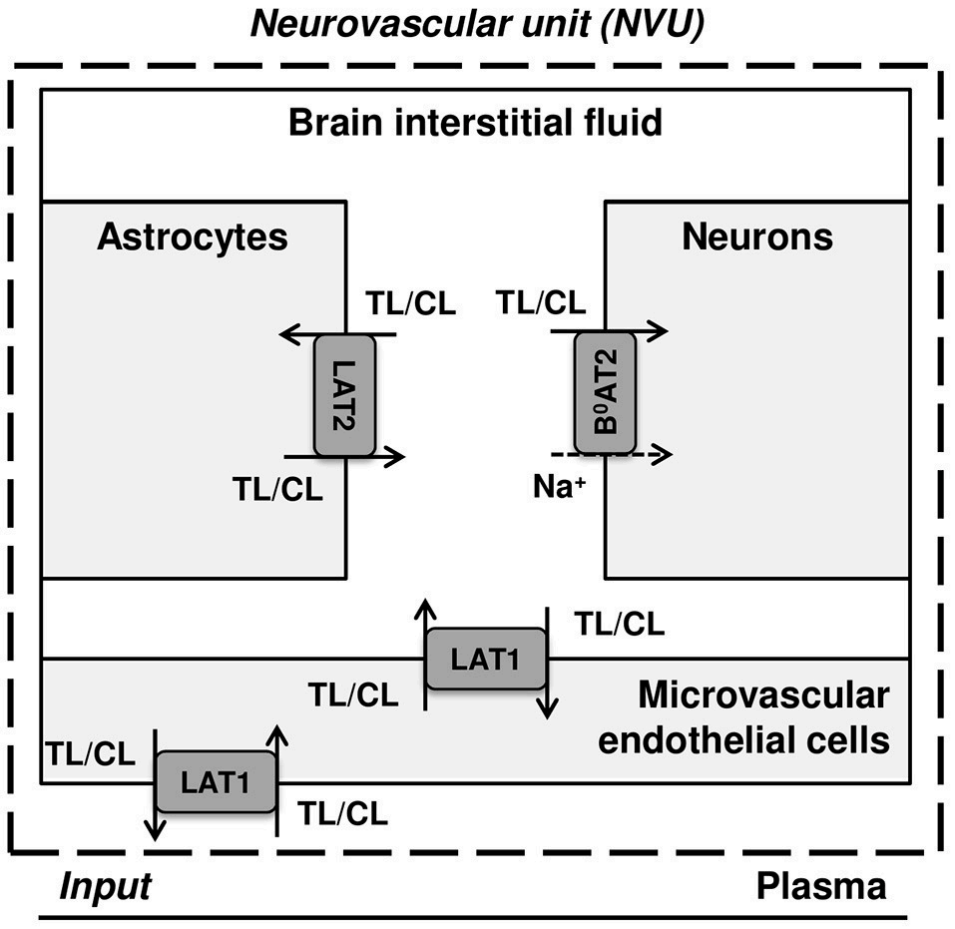
Diagram of the dominant LNAA transporters expressed in cells of the neurovascular unit (NVU). The diagram represents the major compartments of the brain with the dominant NVU carrier-mediated LNAA transport pathways from brain capillary plasma (input) across blood brain barrier (BBB) microvascular endothelial cells (MVEC) into the interstitial fluid (ISF) and from there into astrocytes and neurons. The abbreviations used for the NVU-SLC transporters are LAT1 (SLC7A5) and LAT2 (SLC7A8), both Na^+^-independent large neutral amino acid antiporters, and B^0^AT2 (SLC6A15), a Na^+^-dependent large neutral amino acid symporter. The arrows indicate the transmembrane pathways of LNAAs via these transporters into and out of the NVU cells. TL and CL represent test and competing large neutral amino acids, respectively.

Figure 1 illustrates a simplified model of the adult rat NVU that includes the dominant LNAA transporter of each cell membrane. The NVU-LNAAs have been shown to be transported mainly, but not exclusively, by SLC7A5 (LAT1), SLC6A15 (B^0^AT2), and/or SLC7A8 (LAT2). LAT1 associated with the accessory subunit 4F2hc (SLC3A2) functions as a Na^+^-independent antiporter and plays a dominant role at the luminal and abluminal membranes of the MBECs (Smith et al., 1987; Killian and Chikhale, 2001; Meier et al., 2002). B^0^AT2 is a Na^+^-dependent symporter which has been shown to be the dominant uptake pathway for LNAAs in neurons (Yudkoff et al., 1996b; Bröer et al., 2006; Bak et al., 2012). A number of studies have shown that the Na^+^-independent antiporter LAT2 also associated with 4F2hc is the major mediator of LNAA transport in primary astrocytes (Yudkoff et al., 1996a; Kim et al., 2004; Braun et al., 2011). While it has to be mentioned that comparably high LAT1 mRNA levels have been detected by Zhang et al. (2014) in freshly isolated astrocytes, the functional contribution of this transporter remains unclear (Braun et al., 2011). *In vivo* assays and *in vitro* measurements carried out on freshly isolated cells have shown that the expression of other AATs, such as y^+^LAT2/SLC7A6 (SLC7A6) and ASCT2/SLC1A5 (SLC1A5), is very low in adult brain compared to the aforementioned AATs (Utsunomiya-Tate et al., 1996; Deitmer et al., 2003; Gliddon et al., 2009). Therefore, based on the available evidence in the literature, we consider LAT1, LAT2, and B^0^AT2 to be the predominant LNAA transporters in MBECs, astrocytes and neurons, respectively. Taken together, these transporters co-operate as a highly complex and integrated dynamic system to predominantly control the homeostasis of LNAAs in the brain ISF. For example, LNAAs in the brain ISF can be taken up by B^0^AT2 localized in neurons, and/or they can be exchanged with other LNAAs of astrocytes (mediated by LAT2) and/or be transported back into MBECs and eventually into the bloodstream via LAT1 expressed at the abluminal and luminal membranes of the MBECs (Figure 1).

Among the aforementioned dominant transporters, LAT1 is the central element of the NVU that is involved in the regulation of LNAA homeostasis in the brain ISF. However, despite its importance, its bi-directional kinetic behavior across the BBB has not been characterized yet. We have previously investigated the bi-directional kinetics of LAT1 using the *Xenopus laevis* oocyte expression system, and observed strongly asymmetric bi-directional kinetics (high extra-cellular vs. low intra-cellular binding affinity) (Meier et al., 2002; Verrey, 2003), a finding that has recently been confirmed by reconstitution experiments in proteoliposomes (Napolitano et al., 2015). However, it remains unclear whether this bi-directional asymmetry is dependent on the cell type, and whether it may be influenced by the regulatory/modulatory function of gene products absent in *Xenopus laevis* oocytes and possibly present in other cell types such as MBECs (Meier et al., 2002; Verrey, 2003). *In vivo* tracking of LNAAs from MBECs toward blood plasma and ISF could provide information on the bi-directional kinetic behavior of MBEC LAT1. However, there is currently no suitable *in vivo* technique available to achieve this. While bi-directional uptake and efflux assays using *in vitro* models of MBECs could be used, they may not reflect the *in vivo* situation because of the high sensitivity of the expression level of AATs to culture conditions (Lyck et al., 2009). In addition to the unclarity regarding the bi-directional kinetics of LAT1 in MBECs, the abluminal to luminal expression ratio of LAT1 at the BBB is not well known yet. Only a study carried out in isolated vesicles has characterized the relative expression of LAT1 at the BBB (Sánchez del Pino et al., 1995), but this approach may not reflect the situation *in vivo* (Duelli et al., 2000). Taken together, for the above mentioned reasons, the bi-directional kinetic behavior of LAT1 in MBECs as well as its distribution pattern at the luminal and abluminal membranes of the BBB could so far not be addressed satisfactorily.

To circumvent these limitations, we have developed a robust computational model of LNAA homeostasis in the NVU based on a mathematical description of the nonlinear mechanistic kinetics of the dominant individual NVU-LNAA transporters in conjunction with published *in vivo* LNAA microdialysis (MD) measurements performed in the rat brain ISF upon intraperitoneal administration of L-tyrosine and L-phenylalanine (Bongiovanni et al., 2003, 2010). This has allowed us to explore potential asymmetries of LAT1 bi-directional kinetics and expression in MBECs. Our computations support the hypothesis that MBECs exhibit a functional polarity for LNAAs due to an asymmetry in either bi-directional kinetics and/or expression of LAT1 in MBECs. In addition, we have employed our model to capture changes in LNAA levels in MBEC, astrocytes, and neurons upon perturbations of plasma LNAA concentrations. Finally, we employed the computational model to explain the trans-stimulation of LNAAs upon ISF perfusion of MBEC LAT1 competitive inhibitor.

## Methods:

### Transport model

The NVU is represented by four interacting compartments for MBEC, ISF, astrocytes, and neurons, each with a homogeneous mixture of LNAAs. The plasma conditions are prescribed as dynamic inputs to the NVU (Figure 1). Carrier-mediated transport of LNAAs between the compartments is represented by fluxes dominantly mediated by AATs located at the interface between compartments (Panitchob, 2015; Panitchob et al., 2015, 2016). Following these modeling assumptions, temporal changes in the test LNAA (TL) concentration within the individual NVU compartments are given by

(1) $$\frac{\text{d}{\left[\text{TL}\right]}^{\text{MBEC}}}{\text{dt}}=\frac{1}{{\text{V}}_{\text{MBEC}}}\;\left(\text{f}{\;}_{\text{LAT1},\text{lum}}^{\text{P}\to \text{MBEC}}-\text{f}{\;}_{\text{LAT1},\text{abl}}^{\text{MBEC}\to \text{ISF}}\right)\;,$$

(2) $$\frac{\text{d}{\left[\text{TL}\right]}^{\text{ISF}}}{\text{dt}}=\frac{1}{{\text{V}}_{\text{ISF}}}\;\left(\text{f}{\;}_{\text{LAT1},\text{abl}}^{\text{MBEC}\to \text{ISF}}-\text{f}{\;}_{\text{LAT2}}^{\text{ISF}\to \text{Ast}}-\text{f}{\;}_{{\text{B}}^{0}\text{AT2}}^{\text{ISF}\to \text{Neu}}\right)\;,$$

(3) $$\frac{\text{ d}{\left[\text{TL}\right]}^{\text{Neu}}}{\text{dt}}=\frac{\text{f}{\;}_{{\text{B}}^{0}\text{AT2}}^{\text{ISF}\to \text{Neu}}}{{\text{V}}_{\text{Neu}}},$$

(4) $$\frac{\text{ d}{\left[\text{TL}\right]}^{\text{Ast}}}{\text{dt}}=\frac{\text{f}{\;}_{\text{LAT2}}^{\text{ISF}\to \text{Ast}}}{{\text{V}}_{\text{Ast}}},$$

where [TL]^i^ and V_i_ represent the concentration of the test LNAA in the compartment i and the volume of that compartment, respectively. The carrier-mediated flux of test LNAA from compartment i to compartment j is denoted with ${\text{f}}_{\text{ AAT}}^{\text{i}\to \text{j}}$, and P, MBEC, ISF, Neu, and Ast refer to plasma, microvascular brain endothelial cell, brain interstitial fluid, neuron and astrocyte, respectively. Subscript *lum* and *abl* refer to the luminal and abluminal membranes of the MBEC, respectively.

The fluxes of LNAAs between NVU compartments depend on the mechanism of the individual transporters and their dependence on (or independence of) sodium ions. LAT1 and LAT2 are sodium independent antiporters, while B^0^AT2 functions as a sodium dependent symporter (Meier et al., 2002; Bröer et al., 2006). The fluxes mediated by these transporters are given by Panitchob (2015), Panitchob et al. (2015, 2016), and Pradhan et al. (2013)

(5) $$\text{f}{\;}_{\text{ LAT1},\text{ lum}}^{\text{P}\to \text{MBEC}}=\frac{{\text{2V}}_{\text{max},\text{LAT1},\text{lum},\text{TL}}\;\left({\left[\text{TL}\right]}^{\text{P}}{\left[\text{CL}\right]}^{\text{MBEC}}-{\left[\text{TL}\right]}^{\text{MBEC}}{\left[\text{CL}\right]}^{\text{P}}\right)}{{{\text{K}}_{\text{m},\text{LAT1},\text{TL}}}^{\text{P}}\left({\left[\text{TL+CL}\right]}^{\text{P}}+{\left[\text{TL}+\text{CL}\right]}^{\text{MBEC}}\right)\;+\;\left(\frac{{{\text{K}}_{\text{m},\text{LAT1},\text{TL}}}^{\text{P}}}{{{\text{K}}_{\text{m},\text{LAT1},\text{TL}}}^{\text{MBEC}}}+1\right)\;\left({\left[\text{TL}+\text{CL}\right]}^{\text{P}}\;{\left[\text{TL}+\text{CL}\right]}^{\text{MBEC}}\right)},$$

(6) $$\text{f}{\;}_{\text{ LAT1},\text{ abl}}^{\text{MBEC}\to \text{ISF}}=\frac{{\text{2V}}_{\text{max},\text{LAT1},\text{abl},\text{TL}}\left({\left[\text{TL}\right]}^{\text{MBEC}}{\left[\text{CL}\right]}^{\text{ISF}}-{\left[\text{TL}\right]}^{\text{ISF}}{\left[\text{CL}\right]}^{\text{MBEC}}\right)}{{{\text{K}}_{\text{m},\text{LAT1},\text{TL}}}^{\text{MBEC}}\left({\left[\text{TL}+\text{CL}\right]}^{\text{MBEC}}+{\left[\text{TL}+\text{CL}\right]}^{\text{ISF}}\right)+\left(\frac{{{\text{K}}_{\text{m},\text{LAT1},\text{TL}}}^{\text{MBEC}}}{{{\text{K}}_{\text{m},\text{LAT1},\text{TL}}}^{\text{ISF}}}+1\right)\left({\left[\text{TL}+\text{CL}\right]}^{\text{MBEC}}\;{\left[\text{TL}+\text{CL}\right]}^{\text{ISF}}\right)},$$

(7) $$\text{f}{\;}_{\text{ LAT2}}^{\text{ISF}\to \text{Ast}}=\frac{{\text{2V}}_{\text{max},\text{LAT2},\text{TL}}\;\left({\left[\text{TL}\right]}^{\text{ISF}}{\left[\text{CL}\right]}^{\text{Ast}}-{\left[\text{TL}\right]}^{\text{Ast}}{\left[\text{CL}\right]}^{\text{ISF}}\right)}{{{\text{K}}_{\text{m},\text{LAT2},\text{TL}}}^{\text{ISF}}\left({\left[\text{TL}+\text{CL}\right]}^{\text{ISF}}+{\left[\text{TL}+\text{CL}\right]}^{\text{Ast}}\right)+\left(\frac{{{\text{K}}_{\text{m},\text{LAT2},\text{TL}}}^{\text{ISF}}}{{{\text{K}}_{\text{m},\text{LAT2},\text{TL}}}^{\text{Ast}}}+1\right)\left({\left[\text{TL}+\text{CL}\right]}^{\text{ISF}}\;{\left[\text{TL}+\text{CL}\right]}^{\text{Ast}}\right)},\;$$

(8) f  B0AT2ISF→Neu=2Vmax,B0AT2,TLD(εε′[Na]ISF[Na]Neu([TL]ISF[CL]Neu−[TL]Neu[CL]ISF+ε′ [TL]ISF[Na]ISF                      Km,B0AT2,CLKm,B0AT2,CLNeu−Neuε[TL]Neu[Na]NeuKm,B0AT2,TLKm,NaISF)ISF,            D=[Na]ISF[Na]Neu(ε′ [TL+CL]ISF( [TL+CL]Neu+Km,CL)Neu+ε [TL+CL]Neu( [TL+CL]ISF+Km,B0AT2,TL)ISF)                     +[Na]Km,B0AT2,CLISFKm,B0AT2,NaNeu Neu[TL+CL]ISF(ε′+1)+[Na]Km,B0AT2,TLNeuKm,B0AT2,NaISFISF                      [TL+CL]Neu (ε+1)+Km,B0AT2,TLKm,B0AT2,CLISF([Na]ISF Km,B0AT2,NaNeu +[Na]NeuKm,B0AT2,Na)ISFNeu                    +2Km,B0AT2,TLISFKm,B0AT2,CLNeuKm,B0AT2,NaISFKm,B0AT2,NaNeu,  ε=e(βz FR T Δψ)and ε′=e((β−1)z FR T Δψ ),

where [CL]^i^ represents the concentration in compartment i of LNAAs competing with the test LNAA, and *V*_max,AAT,TL_ and V_max,AAT,CL_ are the maximum transport rates of the AATs for the test and competing LNAA (competitive inhibitors of test LNAA), respectively. In Equation (8), ε and ε′ are the electrical potential-induced biases for forward and backward transport rates, respectively, and Δψ, β, F, z, R, and T represent potential difference, electrical bias constant, Faraday constant, sodium charge, gas constant and absolute temperature, respectively (Pradhan et al., 2013; Panitchob, 2015; Panitchob et al., 2016). ${{\text{K}}_{\text{m},\text{AAT},\text{TL}}}^{\text{i}}$ and ${{\text{K}}_{\text{m},\text{AAT},\text{CL}}}^{\text{i}}\;$are, respectively, the AAT apparent Michaelis-Menten binding constants for the test and competing LNAAs in the presence of competitors. They are determined by Smith and Takasato (1986) and Smith et al. (1987).

(9) $$\begin{array}{l} {{\text{K}}_{\text{m},\text{AAT},\text{TL}}}^{\text{i}}={{\text{K}}_{\text{m},\text{abs},\text{AAT},\text{TL}}}^{\text{i}}(1+\frac{{\left[\text{CL}\right]}^{\text{i}}}{{{\text{K}}_{\text{m},\text{abs},\text{AAT},\text{CL}}}^{\text{i}}}),\\ {{\text{K}}_{\text{m},\text{AAT},\text{CL}}}^{\text{i}}={{\text{K}}_{\text{m},\text{abs},\text{AAT},\text{CL}}}^{\text{i}}\;(1+\frac{{\left[\text{TL}\right]}^{\text{i}}}{{{\text{K}}_{\text{m},\text{abs},\text{AAT},\text{CL}}}^{\text{i}}}), \end{array}$$

where ${{\text{K}}_{\text{m},\text{abs},\text{AAT},\text{TL}}}^{\text{i}}$ and ${{\text{K}}_{\text{m},\text{abs},\text{AAT},\text{CL}}}^{\text{i}}$ are, respectively, the AATs absolute Michaelis-Menten binding constants for test and competing LNAAs in the absence of competitors (Smith and Takasato, 1986; Smith et al., 1987). For simplicity, the competing LNAAs are treated as a single-entity component, representing the overall concentration of the mixture of individual competing LNAAs (Figure 1). The maximum transport rate and the overall absolute Michaelis-Menten binding constant for the competing LNAA, V_max,AAT,CL_ and ${{\text{K}}_{\text{m},\text{abs},\text{AAT},\text{CL}}}^{\text{i}}$, respectively, are given by Thorn (1949) and Cundy et al. (2004):

(10) $$\begin{array}{l} {\text{V}}_{\text{max},\text{AAT},\text{CL}}=\frac{\sum_{\text{k}=1}^{\text{n}}\left(\frac{\;{\text{V}}_{\text{max},\text{AAT},{\text{CL}}_{\text{k}}}\;\left[{\text{CL}}_{\text{k}}\right]}{{\text{K}}_{\text{m},\text{abs},\text{AAT},{\text{CL}}_{\text{k}}}}\right)}{\sum_{\text{k}=1}^{\text{n}}\left(\frac{\;\left[{\text{CL}}_{\text{k}}\right]}{{\text{K}}_{\text{m},\text{abs},\text{AAT},{\text{CL}}_{\text{k}}}}\right)},\\ {{\text{K}}_{\text{m},\text{abs},\text{AAT},\text{CL}}}^{\text{i}}=\frac{\sum_{\text{k}=1}^{\text{n}}\left[{\text{CL}}_{\text{k}}\right]}{\sum_{\text{k}=1}^{\text{n}}\left(\frac{\;\left[{\text{CL}}_{\text{k}}\right]}{{\text{K}}_{\text{m},\text{abs},\text{AAT},{\text{CL}}_{\text{k}}}}\right)}, \end{array}$$

where [CL_k_] and K_m,abs,AAT,CL_k__ represent, respectively, the concentration and the absolute Michaelis-Menten binding constant of the individual competing LNAAs within the considered mixture (see Supplementary Table 1), and where n is the total number of individual competing LNAAs. The MBEC LAT1 bi-directional kinetics are modeled as

(11) $${{\text{K}}_{\text{m},\text{abs},\text{LAT}1}}^{\text{MBEC}}=\;{\text{RK}}_{\text{LAT}1}{{\text{K}}_{\text{m},\text{abs},\text{LAT}1}}^{\text{P}(\text{ISF})},$$

where RK_LAT1_ is the LAT1 bi-directional kinetic constant, which represents the absolute Michaelis-Menten binding constant for LAT1 in MBECs relative to the corresponding value at the outside of MBECs in the ISF and in plasma. The LAT1 expression ratio in MBECs is modeled as

(12) $${\text{V}}_{\text{max},\text{LAT1},\text{abl}}=\;{\text{RE}}_{\text{LAT1}}\;{\text{V}}_{\text{max},\text{LAT1},\text{lum}},$$

where RE_LAT1_ represents the relative ratio for the maximum transport rate of LAT1 at the abluminal membrane of the MBECs to the corresponding value at the luminal membrane. Eqs. (5-12) and Eqs. (1-4) can be combined to describe the intercompartmental rate of change in the concentration of the test LNAAs, $\left(\frac{\text{d}{\left[\text{TL}\right]}^{\text{i}}}{\text{dt}}\right)$, as a system of nonlinear ordinary differential equations of the following general form:

(13) $$\begin{array}{l} \frac{\text{d}{\left[\text{TL}\right]}^{\text{i}}}{\text{dt}}=\text{function }({\left[\text{TL}\right]}^{\text{i}},{\left[\text{CL}\right]}^{\text{i}},\text{Vi },\;{{\text{K}}_{\text{m},\text{AAT},\text{TL}}}^{\text{i}},{{\text{K}}_{\text{m},\text{AAT},\text{CL}}}^{\text{i}},\\ \;{\text{V}}_{\text{max},\text{AAT},\text{TL}},{\text{V}}_{\text{max},\text{AAT},\text{CL}},\;{\text{RK}}_{\text{LAT}1},{\text{RE}}_{\text{LAT}1}) \end{array}$$

The intra-compartmental concentration change rate of the competing LNAAs ($\frac{\text{d}{\left[\text{CL}\right]}^{\text{i}}}{\text{dt}}$) can be formulated similarly. Values for kinetic parameters of individual AATs (K_m,abs,AAT,TL^*i*^_, K_m,abs,AAT,CL^*i*^_, V_max,AAT,TL_ and V_max,AAT,CL_) and volumes of compartments (V_i_) used in Equations (1–13) are listed in Table 1.

**Table 1:** Model input parameters.

	**L-tyrosine^a^**	**L-phenylalanine^b^**		
**Parameters**	**Value**		**Unit**	**References**
**LAT1 (MBEC)**				
${{\text{K}}_{\text{m},\text{abs},\text{LAT}1,\text{TL}}}^{\text{P}\left(\text{ISF}\right)}$	64	11	μM	Smith et al., 1987
V_max,LAT1,lum,TL_	0.175	0.075	μmol/min	Smith et al., 1987; Tilgmann et al., 1992
${{\text{K}}_{\text{m},\text{abs},\text{LAT}1,\text{CL}}}^{\text{P(ISF)}}$	37^c^	52.9^c^	μM	Smith et al., 1987
V_max,LAT1,lum,CL_	0.086^c^	0.129^c^	μmol/min	Smith et al., 1987; Tilgmann et al., 1992
**LAT2 (Astrocyte)**				
${{\text{K}}_{\text{m},\text{abs},\text{LAT}2,\text{TL}}}^{\text{ISF}\left(\text{Ast}\right)}$	294^d^	110.2^d^	μM	Kim et al., 2004
V_max,LAT2,TL_	0.1128	0.1128	μmol/min	Shank and Campbell, 1984; Segawa et al., 1999
${K_{m,abs,LAT2,CL}}^{\text{ISF(Ast)}}$	163.6^c^	185.9^c^	μM	Kim et al., 2004
V_max,LAT2,CL_	0.1452^c^	0.1494^c^	μmol/min	Shank and Campbell, 1984; Segawa et al., 1999
**B**^0^**AT2 (Neuron)**				
${{\text{K}}_{\text{m},\text{abs},\text{B}}^{0}\text{AT}2,\text{TL}}^{\text{ISF(Neu)}}$	NA	1,050	μM	Bröer et al., 2006
$V_{max,B^{0}AT2,TL}$	NA	0.0086	μmol/min	Rao et al., 1995; Bröer et al., 2006
${{\text{K}}_{\text{m},\text{abs},\text{B}}^{0}\text{AT}2,\text{CL}}^{\text{ISF(Neu)}}$	123.5^c^	126.2^c^	μM	Bröer et al., 2006
$V_{max,B^{0}AT2,CL}$	0.0184^c^	0.0186^c^	μmol/min	Rao et al., 1995; Bröer et al., 2006
${\;K_{m,B^{0}AT2,Na}}^{\text{ISF(Neu)}}$	1,050	1,050	μM	Takanaga et al., 2005
ΔΨ	−70	−70	mV	Smith et al., 1981
β	0.6^e^	0.6^e^	mV	Takanaga et al., 2005; Panitchob, 2015
[Na]^ISF^	141	141	mM	Mori et al., 2002
[Na]^Neu^	40	40	mM	Fedoroff and Vernadakis, 1986
**VOLUME**				
V_MBEC_	3.5		μl	Mori et al., 2002; Licinio and Wong, 2009
V_ISF_	352.6		μl	Tilgmann et al., 1992; Syková et al., 2005
V_Ast_	742		μl	Ren et al., 1992; Anderova et al., 2011
V_Neu_	441.7		μl	Ren et al., 1992; Setou et al., 2004; Hosseini-Sharifabad and Nyengaard, 2007

a *In this column, TL and CL represent L-tyrosine and L-tyrosine competing LNAAs, respectively*.

b *In this column, TL and CL represent L-phenylalanine and L-phenylalanine competing LNAAs, respectively*.

c *The kinetic parameters for the mixture of L-tyrosine and L-phenylalanine competing LNAAs are calculated based on Equation 10 (Supplementary Table 1)*.

d *The kinetic parameters are calculated based on Michaelis-Menten equation*.

e *Estimated based on data by Takanaga et al. (2005), Figure 7D. NA (not applicable) specifies the large neutral amino acid was not reported to be a substrate for the transporter. For calculation of Vmax values, the total rat brain weight, volume and protein content are considered 1.81 g (Stewart, 1918), 1,737 μl (Tilgmann et al., 1992) and 105 mg protein/g brain (Banay-Schwartz et al., 1992), respectively*.

### Model initialization and numerical model

To capture the responses of individual NVU compartments (MBEC, ISF, astrocyte, and neuron) to perturbations in plasma LNAA concentration, the baseline (pre-stimulus or pre-injection) state of the system needs to be determined. To this end, we first obtain the steady-state solution of Equation 13 ($\frac{\text{d}{\left[\text{TL}\right]}^{\text{i}}}{\text{dt}}\;=$ $\frac{\text{d}{\left[\text{CL}\right]}^{\text{i}}}{\text{dt}}=\;\text{0}$) by prescribing constant plasma concentrations of LNAAs as NVU system input (Figure 1 and Supplementary Table 2) and solving the resulting system of equations whose unknowns are the baseline LNAA concentrations in the individual compartments. To do so, we are required to initialize the LNAA concentrations in individual NVU compartments. The LNAA concentrations in the ISF, astrocytes, and neurons are initialized according to baseline values reported in the literature (${\left[\text{TL}\right]}_{\text{b}}^{\text{i}}$ and ${\left[\text{CL}\right]}_{\text{b}}^{\text{i}}$) (Supplementary Table 2). Such information is not available for MBECs, however. Therefore, we initialize the corresponding LNAA concentration based on a parametric study obtained with random values of the initial baseline concentration (Supplementary Table 2). It has to be noted that once the LNAA concentrations in the different compartments have been prescribed, the solution of the steady-state problem is constrained in the total amount of LNAAs in the NVU. We examined whether this amount reflects *in vivo* conditions by extrapolating the calculated compartmental LNAA concentrations to the brain as a whole and comparing these values to experimental results reported in Kandera et al. (1968) and Amorini et al. (2017), finding very good agreement (Supplementary Table 2). Once the baseline or pre-stimulus state of the NVU system is determined, we calculate the post-stimulus state of the NVU in response to perturbations of LNAA concentrations in the plasma.

All amino acid transport models were implemented in Matlab (R2015a). To calculate the concentration of LNAAs in the individual NVU compartments (pre- and post-stimulus states), we performed the time integration of Equation 13 using the ode23s function (Bogacki–Shampine method) (Bogacki and Shampine, 1989; Shampine and Reichelt, 1997). The source code from Panitchob (2015) has been used as a starting point for our implementation.

## Results

### Computational model combined with *in vivo* brain ISF measurements support a functional polarity of MBECs

**Figure 2:**
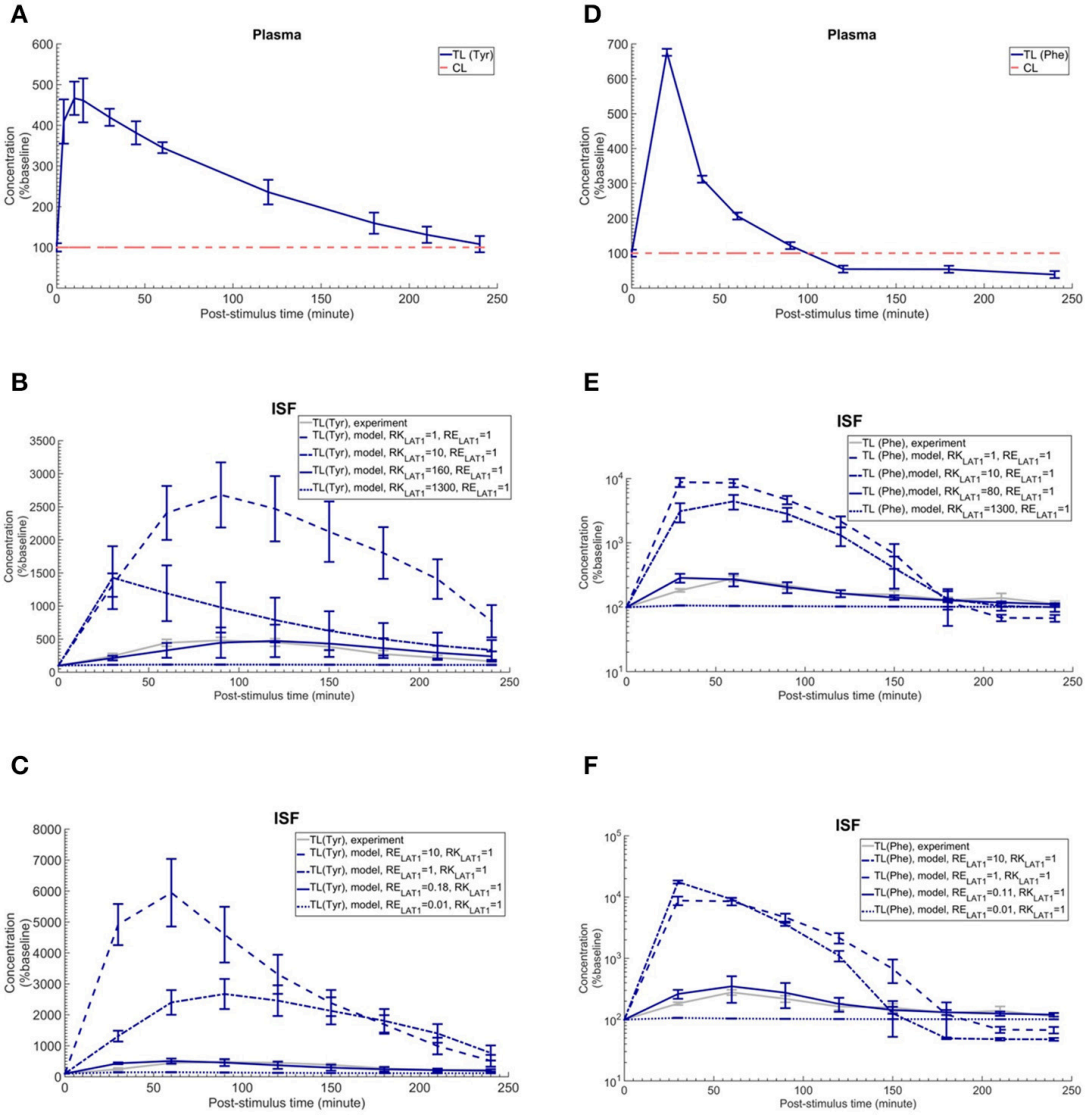
Plasma concentration and corresponding brain ISF concentration response after intraperitoneal injection of L-tyrosine and L-phenylalanine. **(A)** shows the plasma concentration of L-tyrosine (TL) and L-tyrosine competing LNAAs (CL) after intraperitoneal administration of 200 mg/kg L-tyrosine as measured by Bongiovanni et al. (2003) and used as input for the model calculation. **(B,C)** show the experimental data for the L-tyrosine (Tyr) post-stimulus response in the brain ISF, measured in the prefrontal cortex (PFC). **(B)** shows the model calculations for various ratios of the bi-directional kinetic constant of MBEC LAT1 (*RK*_*LAT*1_, Equation 11) with symmetric distribution of LAT1 at both luminal and abluminal membranes of the BBB (RE_LAT1_ = 1). **(C)** shows the model calculations for various abluminal to luminal expression distribution ratios of LAT1 (RE_LAT1_, Equation 12) with symmetric bi-directional kinetics (RK_LAT1_ = 1). The model results and experimental data are represented as percent of the baseline value. In **(A)**, the plasma baseline value for L-tyrosine and L-tyrosine competing LNAAs (constant input) are 112 and 535 μM (Currie et al., 1995; Bongiovanni et al., 2003), respectively. In **(B,C)**, the ISF baseline value for L-tyrosine is 1.0 and 1.1 μM (Supplementary Table 2), respectively. Each experimental data point represents the mean ± SD for three (plasma) and four to eight (ISF) animals (Bongiovanni et al., 2003). In **(A)**, the CL refers to a mixture of L-tyrosine competing LNAAs (L-leucine, L-isoleucine, L-phenylalanine, L-tryptophan, L-valine, L-histidine, and L-methionine). The error bars associated with model calculations indicate standard deviation with respect to concentrations obtained with the nominal model parameter set (see Methods). **(D)** shows the measured plasma concentration of L-phenylalanine (TL) and L-phenylalanine competing LNAAs (CL) after intraperitoneal administration of 200 mg/kg L-phenylalanine as measured by Goldstein (1961) and Bongiovanni et al. (2010). **(E,F)** show the experimental data for the L-phenylalanine (Phe) post-stimulus response in the brain ISF, measured in the prefrontal cortex (PFC) vs. model calculations for different ratios for the bi-directional kinetic constant of MBEC LAT1 (RK_LAT1_, Equation 11), assuming symmetric distribution for LAT1 at luminal and abluminal membranes of the BBB (RE_LAT1_ = 1) and the model calculations for various abluminal to luminal expression distribution ratios of LAT1 (RE_LAT1_, Equation 12), assuming symmetric bi-directional kinetics of MBEC LAT1 (RK_LAT1_ = 1). In **(E,F)**, the ISF baseline value for L-phenylalanine is 0.4 μM (Supplementary Table 2). The data are represented as percent of baseline. In **(D)**, the plasma baseline value for L-phenylalanine and L-phenylalanine competing LNAAs (constant input) are 77 and 562 μM (Currie et al., 1995; Bongiovanni et al., 2003), respectively. In **(D)**, the CL refers to a mixture of LNAAs competing with the test amino acid L-phenylalanine (L-leucine, L-isoleucine, L-tyrosine, L-tryptophan, L-valine, L-histidine, and L-methionine). In **(B–E)**, the differences between the concentrations calculated with the symmetric model (RK_LAT1_ = 1 and RE_LAT1_ = 1) and the experimental measurements are statistically significant at all post-stimulus time points (*p* < 0.001, Supplementary Table 4). In contrast, there is no significant difference between the experimental measurements and the model calculations with RK_LAT1_ = 160 and RE_LAT1_ = 1 **(B)**, RK_LAT1_ = 1 and RE_LAT1_ = 0.18 **(C)**, RK_LAT1_ = 80 and RE_LAT1_ = 1 **(E)** and RK_LAT1_ = 0.11 and RE_LAT1_ = 1 **(F)** with the exception of the 30 min post-stimulus time point in **(C,E,F)** (Supplementary Table 4).

To discriminate, using our new computational model of the NVU, the hypothesized effects of asymmetry on bi-directional kinetics and expression of LAT1 in MBECs (see Introduction), we first searched the literature for kinetic parameters of LNAA transporters of the individual NVU compartments (Table 1). Most carefully measured kinetic parameters of transport at the endothelial barrier reported by Smith et al. (1987) were obtained by using in situ brain perfusion with short uptake times and thus likely represent the kinetics of the first step of LNAAs transport that is into MBECs across their luminal membrane (Bongiovanni et al., 2003, 2010; Pardridge, 2006; Dolgodilina et al., 2015) and are thus not representative of steady-state trans-MBEC transport. Using these kinetic parameters, we first considered the bidirectional kinetics of LAT1 to be symmetric in MBECs (RK_LAT1_ = 1) and also assumed LAT1 to be symmetrically expressed at the luminal and abluminal membranes of MBECs (RE_LAT1_ = 1). Under these assumptions of symmetry, we compared the output of our computational model with *in vivo* measurements made by Bongiovanni et al. (2003). In their study, they had increased the plasma level of L-tyrosine (test LNAA) by intraperitoneal (IP) injection in awake rats and simultaneously measured the post-stimulus response in the brain ISF by microdialysis. Using their measured plasma-stimulus profiles of the test LNAA L-tyrosine ([TL]^P^) and of the L-tyrosine competing LNAAs (competitive inhibitors) ([CL]^P^) as input to the model (Figure 2A, results reported as a percentage of baseline), we calculated the corresponding post-stimulus responses in the brain ISF and found a significant mismatch between the measured and our calculated results which showed a larger excursion due to a much faster transport rate across MBECs (see results of statistical analysis in Figure 2B and Supplementary Table 4).

We then evaluated whether asymmetric bi-directional kinetics of LAT1 in MBECs could explain the slower and less important impact of plasma L-tyrosine perturbation on its ISF concentration observed *in vivo*, compared to our first calculations made assuming symmetric transport properties of LAT1. To this end, we varied the ratio of extracellular to intracellular Michaelis-Menten binding constants of LAT1 in MBECs, named here RK_LAT1_, from 1 (representing the symmetric bi-directional kinetic) to 1300 (highly asymmetric bi-directional kinetics as described for LAT1 in Meier *et al*. (Meier et al., 2002) and considered LAT1 to be symmetrically distributed at the BBB (RE_LAT1_ = 1). We calculated the post-stimulus LNAA concentration response and compared the results with the *in vivo* measurements (shown as percentage of baseline in Figure 2B). Under consideration of asymmetric bi-directional kinetics for LAT1 in MBECs, the numerical results agreed well with *in vivo* experimental data, best for a bi-directional kinetic constant of RK_LAT1_ = 160. Thus, the results obtained with our model support the hypothesis that LAT1 displays a strong asymmetry in bi-directional kinetics in MBECs.

We then evaluated the alternative or complementary hypothesis that an asymmetry of LAT1 expression at the luminal and abluminal membranes of MBECs could explain the observed equilibration kinetics. To this end, we varied the LAT1 expression constant at the BBB (RE_LAT1_) between 0.01 and 10 (representing highly symmetric abluminal to luminal expression ratio) and compared the numerical calculations with the *in vivo* measurements assuming symmetric bi-directional kinetics of the MBEC LAT1 (RK_LAT1_ = 1) (plotted as percentage of baseline in Figure 2C). The error bars associated with model simulations are calculated based on sensitivity studies (see Sensitivity analysis section). In contrast to the symmetric case, the numerical results obtained for asymmetric transporter expression agreed well with *in vivo* experimental data, best for an expression kinetic constant of RE_LAT1_ = 0.18 (see Figure 2C). These results are compatible with the hypothesis of a strong asymmetry in the expression of the LAT1 in MBECs with lower expression at the abluminal membrane. Taken together, the computational model, combined with *in vivo* measurements supports a functional polarity of MBECs with either asymmetry in bi-directional kinetics and/or expression distribution of LAT1 in MBECs.

### Cross-substrate versatility

We next evaluated whether our conclusion on the functional polarity of MBECs described in the previous section depends on the substrate by comparing our calculations with *in vivo* data published by Goldstein (1961) and Bongiovanni et al. (2010) in which the ISF response was measured after IP administration of L-phenylalanine in awake rats (Figure 2F). Just as with the L-tyrosine case, the model failed to reproduce the experimental measurements when assuming symmetric bi-directional kinetics for LAT1 in MBECs, whereas we found a close match between our model calculations and experimental measurements assuming asymmetric LAT1 bi-directional kinetics (best with RK_LAT1_ = 80). Similarly, the numerical results obtained when assuming an asymmetric transporter expression also agreed well with *in vivo* experimental data, best for an expression kinetic constant of RE_LAT1_ = 0.11. Taken together, the comparison of model output with experimental measurements supports the hypothesis that the MBECs show a functional polarity for both L-tyrosine and L-phenylalanine which could be explained by either asymmetric distribution of LAT1 at the luminal and abluminal membranes of the MBECs (lower abluminal expression) and/or a strong asymmetry in its bi-directional kinetics in MBECs (lower intracellular affinity) as previously shown in *Xenopus* oocytes.

To further evaluate the dependence of our results on the asymmetric function of LAT1 suggested for MBECs, we checked whether considering LAT1 as dominant astrocytic AAT instead of LAT2 would modify our conclusion on the functional polarity of the MBECs. Calculations presented in the Supplementary Material (Supplementary Figure 1) showed that this is not the case.

### Calculating the post-stimuli responses in MBECs, astrocytes, and neurons

**Figure 3:**
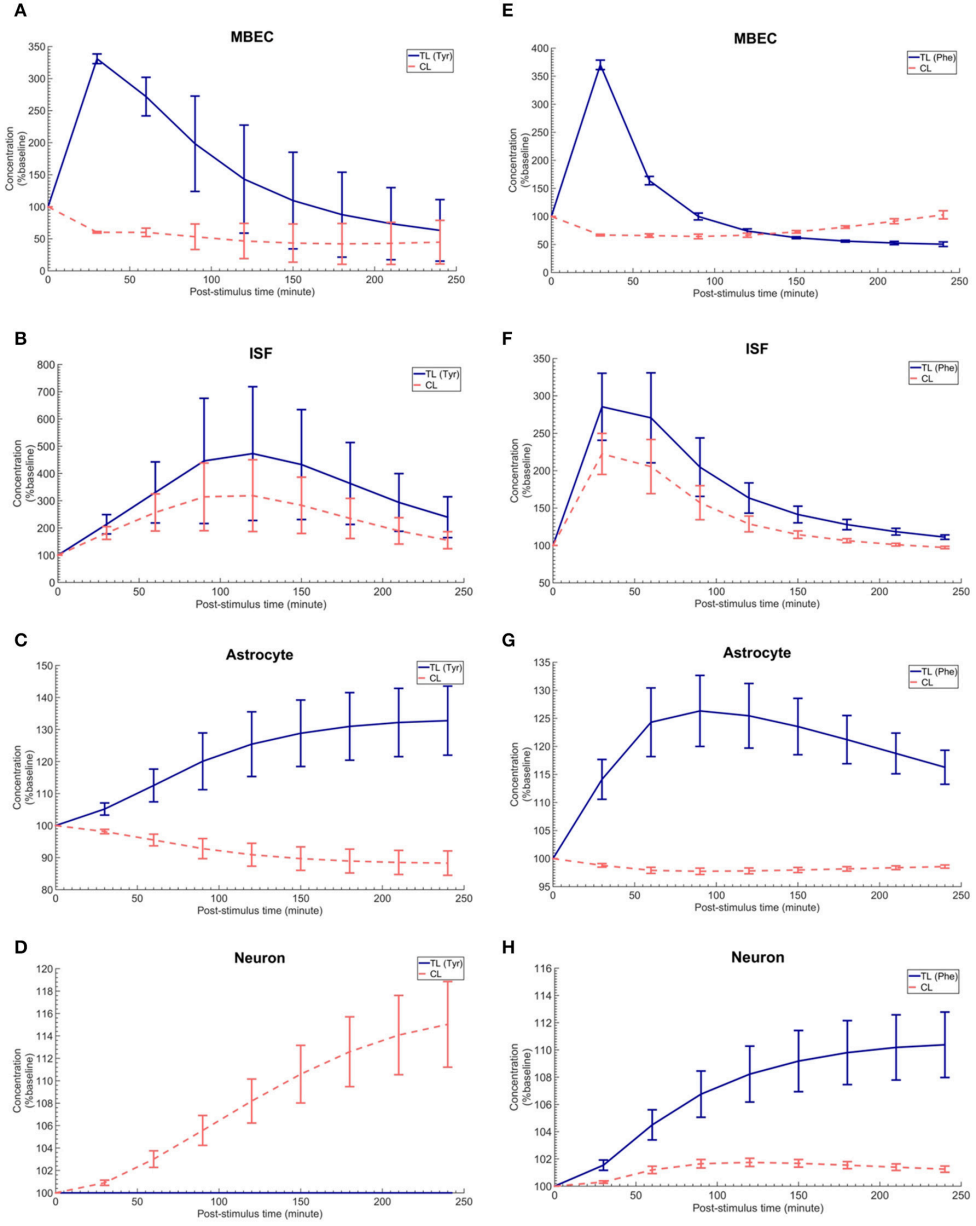
The post-stimulus response in MBECs, ISF, astrocytes and neurons after intraperitoneal administration (IP) of L-tyrosine and L-phenylalanine for asymmetric bi-directional kinetics of LAT1 in MBECs. **(A–H)** show the model calculations for the post-stimulus responses in the NVU individual compartments after IP administration of L-tyrosine (RK_LAT1_ = 160 and RE_LAT1_ = 1) and L-phenylalanine (RK_LAT1_ = 80 and RE_LAT1_ = 1), respectively. The error bars associated with model calculations indicate standard deviation with respect to concentrations obtained with the nominal model parameter set. In **(A–D)**, CL refers to a mixture of L-tyrosine competing LNAAs (L-leucine, L-isoleucine, L-phenylalanine, L-tryptophan, L-valine, L-histidine, and L-methionine). In **(E–H)**, CL indicates a mixture of L-phenylalanine competing LNAAs (L-leucine, L-isoleucine, L-tyrosine, L-tryptophan, L-valine, L-histidine, and L-methionine). The ISF post-stimulus response for TL in **(B,F)** are replotted from Figures 2B,E, respectively. In all panels, the baseline concentration for L-tyrosine, L-tyrosine competing LNAAs, L-phenylalanine and L-phenylalanine competing LNAAs are reported in Supplementary Table 2.

**Figure 4:**
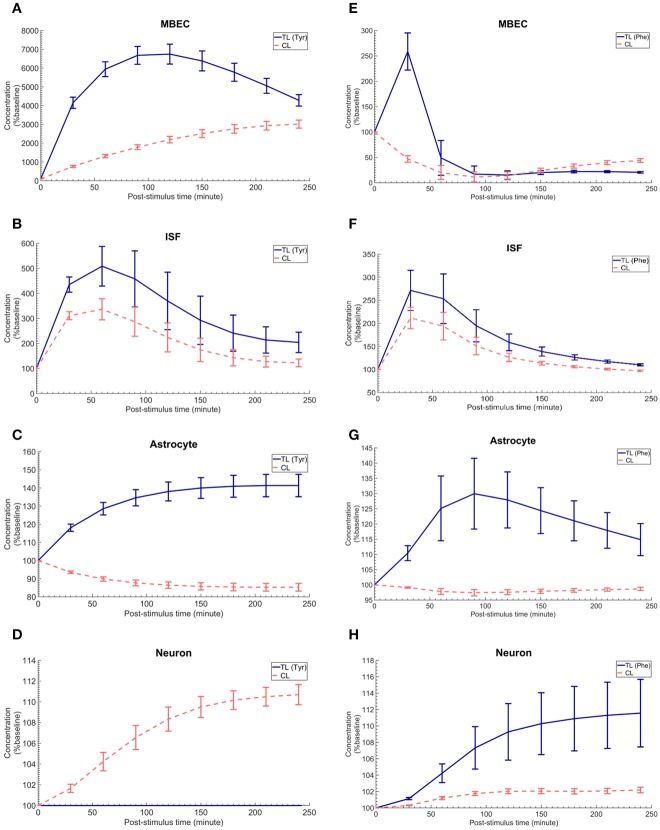
The post-stimulus response in MBECs, ISF, astrocytes and neurons after intraperitoneal administration (IP) of L-tyrosine and L-phenylalanine for asymmetric expression distribution of LAT1 at luminal and abluminal membranes of the BBB. **(A–H)** show the model calculations for the post-stimulus responses in the NVU individual compartments after IP administration of L-tyrosine (RE_LAT1_ = 0.18 and RK_LAT1_ = 1) and L-phenylalanine (RE_LAT1_ = 0.11 and RK_LAT1_ = 1), respectively. The error bars associated with model calculations indicate standard deviation with respect to concentrations obtained with the nominal model parameter set. In **(A–D)**, CL refers to a mixture of L-tyrosine competing LNAAs (L-leucine, L-isoleucine, L-phenylalanine, L-tryptophan, L-valine, L-histidine, and L-methionine). In **(E–H)**, CL indicates a mixture of L-phenylalanine competing LNAAs (L-leucine, L-isoleucine, L-tyrosine, L-tryptophan, L-valine, L-histidine, and L-methionine). The ISF post-stimulus response for TL in **B,F** are replotted from Figures 2C,F. In all panels, the baseline concentration for L-tyrosine, L-tyrosine competing LNAAs, L-phenylalanine and L-phenylalanine competing LNAAs are reported in Supplementary Table 2.

The *in vivo* standard methods have so far not been able to address the effects of plasma LNAA perturbations on the dynamics of LNAA concentrations in individual NVU compartments. To close this gap, we employed the computational model considering either an asymmetry in bi-directional kinetics of LAT1 or an asymmetry in the expression pattern of LAT1 in MBECs as determined for the best cases in Figure 2. The dynamic responses of L-tyrosine (TL) and L-tyrosine competing LNAAs (CL) and of L-Phenylalanine (TL) and its competitor LNAA (CL) in MBECs, ISF, astrocytes, and neurons are shown in Figures 3, 4 for asymmetric bi-directional kinetics and asymmetric expression of LAT1, respectively. The same plasma perturbations of the test LNAAs (L-tyrosine or L-phenylalanine) used also for Figure 2 are shown to first propagate into the MBECs (Figures 3A,E, 4A,E). The dynamics of this propagation depend on the competitions between the test and competing LNAAs through MBEC LAT1 and the kinetics for each substrate. Since MBEC LAT1 functions as an antiporter, the elevated level of the test LNAA in the MBECs leads to an initial reduction in the MBEC level of the competing LNAAs (Figures 3A,E, 4A,E). Subsequently, the test and competing LNAAs compete for efflux via LAT1 across the abluminal membrane of the BBB MBECs and eventually gain entry into the brain ISF in exchange for competing LNAA of the ISF (Figures 3B,F, 4B,F). The observed delayed response in the concentration of the test LNAAs in brain ISF in response to the plasma perturbations is mainly due the low inter-endothelial affinity of LAT1 (Figure 3B) and/or a low expression of LAT1 at the abluminal membrane of the BBB (Figure 4B) both of which would strongly limit the trans-endothelial transport of LNAAs across the BBB. Once the test (and competing) LNAAs enter the brain ISF, they are differentially co-transported together with sodium ions into neurons via B^0^AT2 (Figures 3D,H, 4D,H) and exchanged back into the MBECs (Figures 3A,E, 4A,E) and astrocytes (Figures 3C,G, 4C,G) via LAT1 and LAT2, respectively. The rate of these transports depends on LNAA concentration, on that of competitor LNAAs and on the kinetics of the transporters expressed at the interface to the other NVU compartments (Table 1). For example, LNAA transport from ISF to MBECs is comparably low due to the relatively low concentration of the LNAAs compared to their Michaelis-Menten binding affinities (Table 1 and Supplementary Table 2). As shown in Figures 3C,G, 4C,G, astrocytic elevation of the test LNAAs is associated with the reduction of intracellular competing LNAAs. This behavior is due to the exchange mechanism of LAT2 localized at the membrane of astrocytes. In Figures 3D, 4D, while L-tyrosine transport is shown not to be mediated by B^0^AT2 (Table 1), this LNAA could nonetheless be transported to some extent into neurons by other, less expressed transporters (see Discussion section). Taken together, the difference between the response of L-tyrosine and L-phenylalanine results from various factors such as their differing original perturbation dynamics in plasma (Figures 2A,D) and the transport kinetics differences of the NVU-AATs for these substrates and their competitors (Equation 13 and Table 1).

### The trans-stimulation of the test LNAA uptake across the BBB upon ISF perfusion with a LAT1 competitive inhibitor

**Figure 5:**
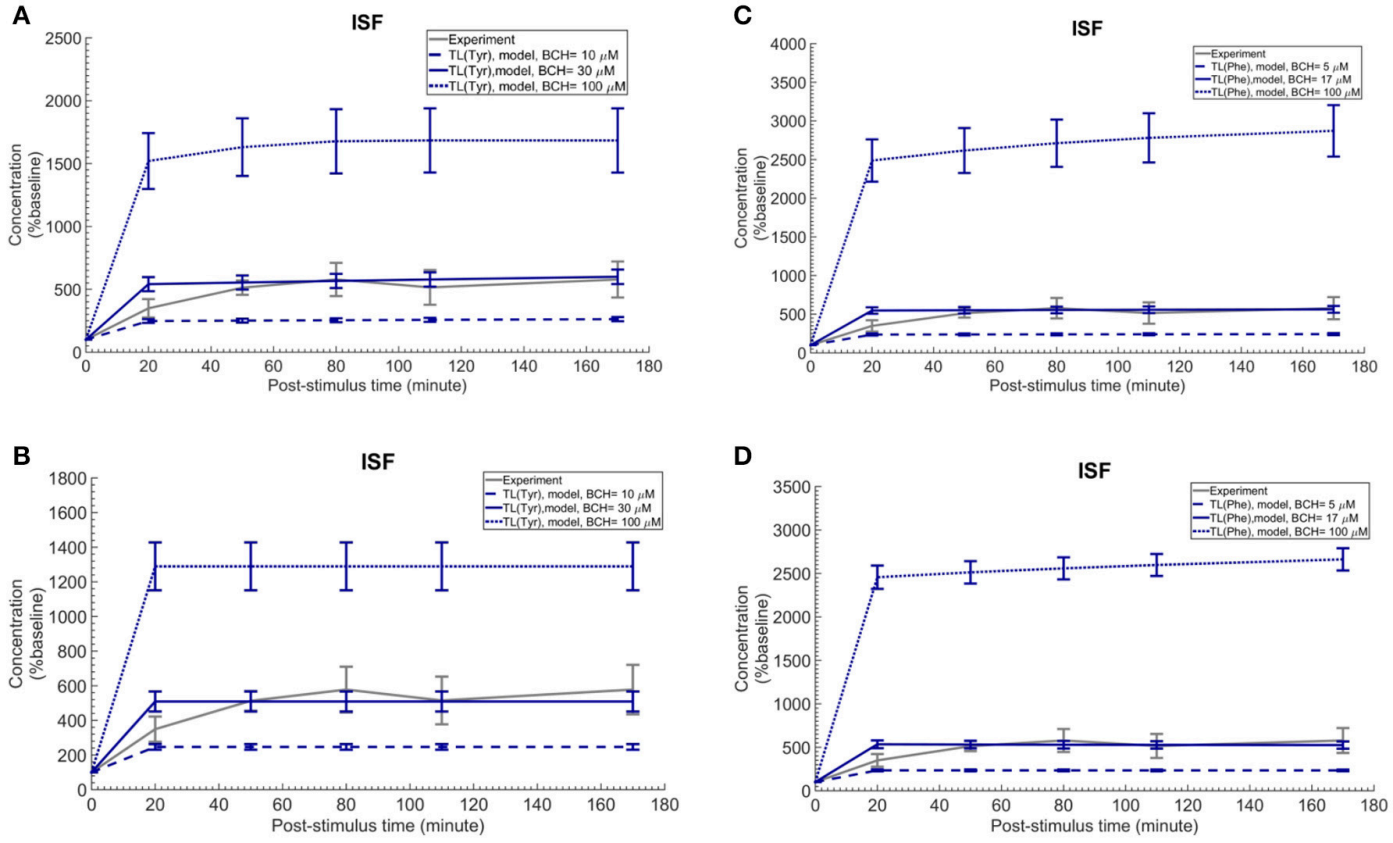
Trans-stimulation of the test LNAA uptake across the BBB during ISF perfusion with BCH. This figure shows the ISF concentration of the test LNAAs during ISF perfusion with 2-aminobicyclo-(2,2,1)-heptane-2-carboxylic acid (BCH) started at time zero. In all panels, the experimental data are measured by Dolgodilina et al. (2015) for trans- stimulation of test LNAA (L-valine) during 170 min continues ISF perfusion with 20 mM BCH into a group of freely moving mice (four animals). **(A,B)** show the model calculations for L-tyrosine trans-stimulations upon perfusion of BCH with different global concentration levels. In **(A,B)**, the bidirectional kinetic constant and the expression ratio of LAT1 are considered, (RK_LAT1_ = 160, RE_LAT1_ = 1) and (RK_LAT1_ = 1, RE_LAT1_ = 0.18), respectively. **(C,D)** show the model calculations for L-phenylalanine trans-stimulations during perfusion of BCH with different global concentration levels in the entire brain ISF compartment. In **(A,B)**, the bi-directional kinetic constant and the expression ratio of LAT1 are considered, (RK_LAT1_ = 80, RE_LAT1_ = 1) and (RK_LAT1_ = 1, RE_LAT1_ = 0.11), respectively. The model simulations and the experimental data are represented as percent of the baseline value. The error bars associated with model calculations indicate standard deviation with respect to concentrations obtained with the nominal model parameter set. For all panels, the calculated baseline concentrations of the test LNAAs are reported in Supplementary Table 2. The differences between the experimental measurements and model calculations with BCH = 10 and 100 μM **(A,B)** as well as BCH = 5 and 100 μM **(C,D)** are statistically significant at all post-stimulus time points (*p* < 0.001, Supplementary Table 4). In contrast, model calculations with BCH = 30 μM **(A,B)** and BCH = 17 μM **(C,D)** are not significantly different from the experimental measurements with the exception of the 20 min post-stimulus time point (Supplementary Table 4).

Finally, we employed the established computational model to investigate the induced effects of brain ISF perfusion with 2-aminobicyclo-(2,2,1)-heptane-2-carboxylic acid (BCH, a transported competitive inhibitor of LAT1 and LAT2) on the dynamics of test LNAAs in the brain ISF (Taslimifar et al., 2017). We have shown recently that continues perfusion of 20 mM BCH [~ 2 mM local concentration near the perfusion probe (Dolgodilina et al., 2015)] into the brain ISF of freely moving mice trans-stimulates the LAT1 functions at the BBB and consequently changes the dynamics of LNAAs in the brain ISF in exchange for the perfused BCH (Dolgodilina et al., 2015). To mimic the experimental conditions, we have prescribed the brain ISF concentration of BCH as constant input to the model (considering BCH as competing LNAA (CL) with the same kinetics Taslimifar et al., 2017) and consequently calculated the post-stimulus responses in the concentration of test LNAAs. Considering the fact that measuring the global concentrations of BCH in the entire brain ISF compartment is experimentally challenging, we compared the numerical calculations with the *in vivo* measurements for different values for the global concentrations of BCH which are much lower than the local BCH concentrations near the probes. The computational results for the dynamic changes of L-tyrosine and L-phenylalanine calculated using the kinetic and expression ratios of LAT1 (RK_LAT1_ and RE_LAT1_) determined above are plotted in Figures 5A,B, respectively as percentage of the baseline. The error bars associated with model simulations are calculated based on sensitivity studies described below. As shown in all panels, the elevation of perfused BCH concentration leads to increased stimulation of the transport of test LNAAs into the brain ISF which is due to the stimulated exchange of the perfused BCH with the test LNAAs via MBEC LAT1 and astrocyte LAT2 (trans-stimulation of efflux from these cells). The model calculations for the stimulated test LNAAs eventually reach a plateau consistent with our previous experimental observations. The best match between model and experimental measurements was observed for global BCH concentrations of 17–30 μM in the brain ISF. It has to be noted that our model, by assuming a homogenous mixture of LNAAs within the individual NVU compartments, disregards the delayed diffusion time of the perfused BCH from the probe site into the ISF which already explains the initial difference between model calculations and experimental measurements in all panels (see Discussion).

### Sensitivity analysis and statistical testing

We assessed the sensitivity of the reported results with respect to the choice of literature-reported values of model parameters. To accomplish this goal, we simultaneously varied the nominal model input parameters (Michaelis-Menten binding constant, maximum transport rate of AATs Table 1 and the initialized baseline concentration of LNAAs in individual compartments Supplementary Table 2) within realistic bounds (±20% for each parameter), and then assessed the model output for 100 random parameter sets. The results of the sensitivity analysis are presented in Supplementary Tables 2, 3, as well as in Figures 2–5 and Supplementary Figure 1, where error bars indicate standard deviation of the computed concentrations from those obtained under nominal parameter conditions. We then assessed whether differences in the set of calculated and experimentally measured concentration profiles are statistically significant. To this end, we performed at each post-stimulus time point Student's unpaired t-test with Holm-Sidak correction for multiple comparisons using GraphPad Prism 5.0 (GraphPad Software, USA). *P* < 0.01 were considered indicative of statistical significance (see Supplementary Table 4 for test results).

## Discussion

In this study, using a computational model and experimental input data, we obtained results that strongly suggest a functional polarity of MBECs for the trans-endothelial transport of LNAAs, and characterized a potential strong asymmetry in bi-directional kinetics and/or an asymmetry in membrane expression of MBEC LAT1, which could so far not be addressed with current standard *in vitro* and *in vivo* methods. The robust computational model of NVU-LNAA transport we have built and used in this study is based on the fluxes mediated by the respective dominant transporters expressed in MBECs, astrocytes and neurons, namely LAT1, LAT2 and B^0^AT2. This allowed us to test different symmetric and asymmetric hypotheses about the bi-directional kinetics and/or the expression of LAT1 in MBECs. The comparison of our computational results with published *in vivo* microdialysis measurements obtained in rat brain supports the hypothesis that MBEC LAT1 either exhibits strong asymmetric bi-directional kinetics for LNAAs (lower affinity inside the MBECs) and/or is asymmetrically expressed at the BBB (lower expression at the abluminal membrane of the BBB). This observation is shown to be independent of the substrate considered (i.e. L-tyrosine and L-phenylalanine).

After the characterization of the functional polarity of MBECs, we aimed at understanding the response of the individual NVU cells to IP administration of LNAAs, which has not been addressed so far by *in vivo* standard methods. To accomplish this, we employed the computational model to calculate the changes in the concentrations of NVU-LNAAs in response to IP administration of L-tyrosine and L-phenylalanine, considering asymmetry in either bi-directional kinetics and/or expression distribution of LAT1 in MBECs. We thereby captured the interactive dynamics of LNAAs as they traverse the blood-brain barrier (BBB) from the capillary lumen into the brain interstitial fluid and from there eventually into astrocytes and/or neurons. Finally, we employed the model to explain also the trans-stimulation of LNAA uptake across the BBB upon ISF perfusion with BCH, a competitive inhibitor of LAT1.

LAT1 is the primary entry way to the brain for a broad range of the essential LNNAs and their analogs, such as L-DOPA, gabapentin and L-melphalan (Killian and Chikhale, 2001; Cundy et al., 2004; Rautio et al., 2013). Hence, our finding of its asymmetric kinetics and/or expression in MBECs provides novel insight that may help advance our understanding of LAT1-mediated prodrug delivery (i.e., meta-substituted phenylalanine prodrugs) to the brain (Gynther et al., 2008; Rautio et al., 2008; Peura et al., 2011). In addition, our computational model could be employed to provide insight into the amino acid transport processes in brain disorders associated with perturbations of LNAAs in the plasma, e.g., phenylketonuria (PKU) or Maple syrup urine disease (MSUD). This could be achieved by using the plasma LNAA perturbations observed in patients as input to the model to calculate the corresponding responses in the NVU-LNAA concentrations, which are challenging to measure experimentally (Dixon et al., 2015).

We note a number of simplifying assumptions made for the development of our computational model. For instance, the assumption of a homogenous mixture of LNAAs within the individual NVU compartments disregards the local differences in the intra-compartmental concentration of LNAAs. In reality, however, regional distribution of amino acids in NVU compartments may affect the binding of LNAAs to the corresponding transporters, and therefore also the local transport fluxes. Moreover, we have considered competitive LNAAs as a single entity rather than accounting one by one each individual competitor for the transport of L-phenylalanine or L-tyrosine, such as L-leucine, L-tryptophan and others (Supplementary Table 1). This assumption, however, has already been experimentally validated for multi-substrate enzymatic reactions (Alberty, 1956). In addition, we focused on a single carrier per NVU compartment membrane, specifically on the antiporters (obligatory exchangers) LAT1 and LAT2 for MBECs and astrocytes, respectively, and a symporter (cotransporter B0AT2) for neurons, not taking into consideration diffusive pathways which have been shown, however, to be of lesser importance for LNAAs in the NVU (Smith and Takasato, 1986). Moreover, we did not include LNAA metabolism, which is not completely known and understood in the CNS (Sperringer et al., 2017; Yudkoff, 2017). However, it has been shown that the brain metabolic fluxes of LNAAs (such as L-phenylalanine, L-histidine, etc.) are small compared to the carrier-mediated fluxes (Sadasivudu and Lajtha, 1970). Additionally, our model relies on literature-reported parameter values, which are inevitably associated with the reported uncertainty. Nevertheless, our sensitivity analysis has shown that the conclusions drawn in this study hold within reasonable parameter variations. Furthermore, it has to be pointed out that the established computational model takes only into account the interactions between the dominant NVU-LNAA transporters mentioned above and disregards the contribution of other transporters, such as for instance y^+^LAT2 and ASCT2, which have been shown to be expressed in adult brains, though at a lower level (Utsunomiya-Tate et al., 1996; Deitmer et al., 2003; Gliddon et al., 2009). Beyond that, it has to be highlighted that the structure and function of many (SLC) transporters have yet to be fully characterized and that some of them may also transport LNAAs, such that further research is required (Rautio et al., 2013). The contribution of newly discovered NVU transporters could then be included in the computational model upon sufficient characterization of their kinetics.

## Summary

We have characterized a functional polarity for MBECs which are the key NVU element for the control of LNAA homeostasis in the brain ISF. For this purpose, we have developed a robust computational model of NVU-LNAA homeostasis and combined it with published *in vivo* measurements obtained in rat brain. We have shown that either strong asymmetrical bi-directional kinetics of LAT1 in MBECs and/or an asymmetric distribution of LAT1 at both membranes of MBECs is required to reproduce available *in vivo* measurements. This conclusion is strengthened by the fact that it is supported by data obtained for two tested LNAAs, L-tyrosine and L-phenylalanine. Important characteristics of LAT1 function in MBECs have not been tested satisfactorily up to now by experimental means. In addition, based on our findings on the functional polarity of MBECs, we employed our computational model to investigate the dynamic behavior of LNAAs in astrocytes and neurons in response to IP-administered L-tyrosine and L-phenylalanine, values which are challenging to determine experimentally. Finally, we used the model to explain the trans-stimulation of LNAA uptake across the BBB upon ISF perfusion with a LAT1 competitive inhibitor. While we employed our computational platform to answer fundamental physiological questions about homeostatic regulation of LNAAs in the NVU, it could also be used to test strategies designed to improve the treatment and management of LNAA-related brain disorders.

## Author contributions

MT implemented the computational model and performed the calculations with SB. FV and VK contributed equally to this article and directed the research. All authors conceived and designed the study, analyzed the data, wrote the manuscript and approved the final version.

### Conflict of interest statement

The authors declare that the research was conducted in the absence of any commercial or financial relationships that could be construed as a potential conflict of interest.
