# Supplementary material for: Functional Polarity of Microvascular Brain Endothelial Cells Supported by Neurovascular Unit Computational Model of Large Neutral Amino Acid Homeostasis
Source: Front Physiol. 2018 Mar 13;9:171. doi: 10.3389/fphys.2018.00171 (PMC5859092; doi:10.3389/fphys.2018.00171)
Supplement: Supplementary file 1 [file DataSheet1.pdf]

Supplementary Material

Functional polarity of microvascular brain endothelial cells supported by neurovascular unit computational model of large neutral amino acid homeostasis

Mehdi Taslimifar[1,2], Stefano Buoso[1,3], Francois Verrey[2,4,5†], Vartan Kurtcuoglu[1,4,5,6*†]

[1] The Interface Group, Institute of Physiology, University of Zurich, Switzerland;

[2] Epithelial Transport Group, Institute of Physiology, University of Zurich, Switzerland;

[3] Institute for Diagnostic and Interventional Radiology, Zurich University Hospital, Zurich, Switzerland;

[4] Zurich Center for Integrative Human Physiology, University of Zurich, Switzerland;

[5] National Center of Competence in Research, Kidney.CH, Switzerland;

[6] Neuroscience Center Zurich, University of Zurich, Zurich, Switzerland.

[†] contributed equally.

*** Correspondence:** Vartan Kurtcuoglu

Institute of Physiology

University of Zürich

Winterthurerstrasse 190,

CH-8057 Zürich, Switzerland

Telephone: +41 (0) 44 635 50 55

Email: vartan.kurtcuoglu@uzh.ch

1 Supplementary Tables

Supplementary Table 1: The kinetic parameters of the dominant transporters and concentration of individual LNAAs in the different brain compartments

	Kinetic parameters						Concentration		
	**LAT1 (MBEC)**	**LAT2 (Astrocyte)**	**B^0AT2 (Neuron)**	**LAT1 (MBEC)**	**LAT2 (Astrocyte)**	**B^0AT2[e] (Neuron)**	**Plasma**	**ISF**	**Astrocyte and neuron[f]**
	$K_{m,abs}$ (µM)			V_{max} (µmol/min)			Concentration (µM)		
**L-phenylalanine**	11 (Smith et al., 1987)	110.2[a] (Kim et al., 2004)	1050 (Bröer et al., 2006)	0.075 (Smith et al., 1987; Tilgmann et al., 1992)	0.1128 (Shank and Campbell, 1984; Segawa et al., 1999)	0.0193 (Rao et al., 1995; Bröer et al., 2006)	77 (Currie et al., 1995)	0.73 (Currie et al., 1995)	52.1 (Kandera et al., 1968)
**L-valine**	210 (Smith et al., 1987)	110.2[a] (Kim et al., 2004)	190 (Yudkoff et al., 1996b; Bak et al., 2012)	0.089 (Smith et al., 1987; Tilgmann et al., 1992)	0.1427 (Shank and Campbell, 1984; Segawa et al., 1999)	0.0193 (Rao et al., 1995; Bröer et al., 2006)	211 (Currie et al., 1995)	1.22 (Currie et al., 1995)	86.5 (Kandera et al., 1968)
**L-leucine**	29 (Smith et al., 1987)	224.2 (Kim et al., 2004)	81 (Bröer et al., 2006)	0.107 (Smith et al., 1987; Tilgmann et al., 1992)	0.184 (Shank and Campbell, 1984; Segawa et al., 1999)	0.0193 (Rao et al., 1995; Bröer et al., 2006)	149 (Currie et al., 1995)	1.35 (Currie et al., 1995)	66.7 (Kandera et al., 1968)
**L-isoleucine**	56 (Smith et al., 1987)	139.2[a] (Kim et al., 2004)	58 (Bröer et al., 2006)	0.109 (Smith et al., 1987; Tilgmann et al., 1992)	0.131 (Shank and Campbell, 1984; Segawa et al., 1999)	0.0154 (Rao et al., 1995; Bröer et al., 2006)	102 (Currie et al., 1995)	0.77 (Currie et al., 1995)	26.1 (Kandera et al., 1968)
**L-histidine**	100 (Smith et al., 1987)	327[a] (Kim et al., 2004)	NA	0.111 (Smith et al., 1987; Tilgmann et al., 1992)	0.173 (Shank and Campbell, 1984; Segawa et al., 1999)	NA	52 (Currie et al., 1995)	1.12 (Currie et al., 1995)	68.8 (Kandera et al., 1968)
**L-tyrosine**	64 (Smith et al., 1987)	294[a] (Kim et al., 2004)	NA	0.175 (Smith et al., 1987; Tilgmann et al., 1992)	0.113 (Shank and Campbell, 1984; Segawa et al., 1999)	NA	112 (Bongiovanni et al., 2003)	0.76 (Currie et al., 1995)	76.1 (Kandera et al., 1968)
**L-tryptophan**	15 (Smith et al., 1987)	110.2[a] (Kim et al., 2004)	NA	0.099 (Smith et al., 1987; Tilgmann et al., 1992)	0.131 (Shank and Campbell, 1984; Segawa et al., 1999)	NA	17 (Currie et al., 1995)	0.15 (Currie et al., 1995)	13.5 (Amorini et al., 2017)
**L-methionine**	40 (Smith et al., 1987)	430.7[a] (Kim et al., 2004)	40 (Bröer et al., 2006)	0.046 (Smith et al., 1987; Tilgmann et al., 1992)	0.156 (Shank and Campbell, 1984; Segawa et al., 1999)	0.0193 (Rao et al., 1995; Bröer et al., 2006)	96 (Currie et al., 1995)	0.37 (Currie et al., 1995)	38.6 (Kandera et al., 1968)
**CL[bc]**	37	163.6	123.5	0.0859	0.1452	0.0193	704	5.7	352.2
**CL[bd]**	52.9	185.9	126.2	0.0973	0.1494	0.0193	739	5.7	376.2

[a]The values are calculated based on Michaelis-Menten equation (Segel, 1975; Kim et al., 2004) .[b]The kinetic parameters for the mixture of competing LNAAs are calculated based on Eq.10. [c] In this row, CL represent the mixture of L-tyrosine competing LNAAs (mixture of L-leucine, L-isoleucine, L-phenylalanine, L-tryptophan, L-valine, L-histidine and L-methionine). [d]In this row, CL represent the kinetic parameters for the mixture of phenylalanine competing LNAAs (mixture of L-leucine, L-isoleucine, L-tyrosine, L-tryptophan, L-valine, L-histidine and L-methionine). [e]The volume of astrocyte is considered 742 µl (Ren et al., 1992; Anderova et al., 2011) (Table 1). [e]The reported Vmax values for individual LNAAs are based on the Leucine measurements. [f] The concentration of LNAAs in the neuron and astrocyte compartments are based on measurements in the brain tissue (Shank and Campbell, 1984). NA (not applicable) specifies that the large neutral amino acid was not reported to be a substrate for the transporter. For calculation of V_{max} values, the total rat brain weight, volume and protein content and the volume of astrocyte and neuron are considered 1.81 g (Stewart, 1918), 1737 µl, 105 mg protein/g brain (Banay-Schwartz et al., 1992), 742 µl (Ren et al., 1992; Anderova et al., 2011) and 441.7 µl (Ren et al., 1992; Setou et al., 2004; Hosseini-Sharifabad and Nyengaard, 2007) (Table 1), respectively.

Supplementary Table 2: The initialization and model calculations for the baseline concentration of LNAAs in the individual NVU compartments.

	L-tyrosine[a]			L-phenylalanine[b]			
**Parameter**	**Initialized baseline concentration[c]**	**Calculated baseline concentration**		**Initialized baseline concentration[c]**	**Calculated baseline concentration**		**Unit**
Microvascular brain endothelial cell[d]							
$[TL]_b^{MBEC}$	[0-76.1] (Kandera et al., 1968)	274.0 ± 20.9	16.6±1.1	[0-52.1] (Kandera et al., 1968)	146.8 ± 24.6	106.5 ± 13.6	µM
$[CL]_b^{MBEC}$	[0-352.2] (Kandera et al., 1968; Amorini et al., 2017)	1722.2 ± 131.5	104.5±6.6	[0-376.2] (Kandera et al., 1968; Amorini et al., 2017)	1408.8 ± 235.7	1022.4 ±130.7	µM
Brain interstitial fluid[e]							
$[TL]_b^{ISF}$	0.8 (Currie et al., 1995)	1.0±0.1	1.2±0.1	0.7 (Currie et al., 1995)	0.4 ± 0.03	0.4 ± 0.03	µM
$[CL]_b^{ISF}$	5.7 (Currie et al., 1995)	6.5±0.9	7.3±0.9	5.7 (Currie et al., 1995)	3.8 ± 0.3	3.8 ± 0.3	µM
Astrocyte[f]							
$[TL]_b^{Ast}$	76.1 (Kandera et al., 1968)	61.5 ± 7.0	66.1±6.7	52.1 (Kandera et al., 1968)	40.6 ± 4.8	40.2 ± 4.6	µM
$[CL]_b^{Ast}$	352.2 (Kandera et al., 1968)	386.5± 43.7	415.5±41.8	376.2 (Kandera et al., 1968; Amorini et al., 2017)	389.7 ± 46.0	385.7 ± 44	µM
Neuron[f]							
$[TL]_b^{Neu}$	76.1 (Kandera et al., 1968)	75.1 ± 9.0	77.1±9.3	52.1 (Kandera et al., 1968)	53.4 ± 7.0	54.3 ± 6.0	µM
$[CL]_b^{Neu}$	352.2 (Kandera et al., 1968; Amorini et al., 2017)	316.6 ± 41.4	354.8±41.3	376.2 (Kandera et al., 1968; Amorini et al., 2017)	368.7 ± 46.9	375.2 ± 39.8	µM
The bi-directional and expression constant of MBEC LAT1							
RK_{LAT1}	-	160	1	-	80	1	-
RE_{LAT1}	-	1	0.18	-	1	0.11	-

The standard deviations associated with the calculated baseline concentrations are estimated based on the sensitivity studies (Sensitivity analysis section).[a]In this column, TL and CL represent tyrosine and tyrosine competing LNAAs, respectively. For the calculations of baseline concentrations, the plasma concentration of TL and CL are considered as constant input, fixed at 112 (Bongiovanni et al., 2003) and 704 (Currie et al., 1995) µM, respectively (Suppl. Table 1). [b]In this column, TL and CL represent phenylalanine and phenylalanine-competing LNAAs, respectively. For the calculations of baseline concentrations, the plasma concentration of TL and CL are defined as constant input, fixed at 77 (Currie et al., 1995) and 739 (Currie et al., 1995; Bongiovanni et al., 2003) µM, respectively (Suppl. Table 1). [C]The baseline initialization for TL and CL are based on values reported in Suppl. Table 1. [d]The microvascular brain endothelial cells are initialized randomly as the values were not available. The specified range is based on the total concentration in the brain tissue [e]The micro dialysis samples are collected from rat lateral hypothalamus. [f]The initialization for the neuron and astrocyte compartments are based on the measured baseline concentrations of LNAAs in brain tissue (Suppl. Table 1) (Shank and Campbell, 1984).

2 Evaluation of brain ISF post-stimuli responses under the assumption of LAT1 as the dominant LNAA transporter in astrocytes

As mentioned in the Introduction section, several studies have shown that LAT2 is the dominant LNAA transporter in primary astrocyte cells (Yudkoff et al., 1996a; Kim et al., 2004; Braun et al., 2011). However, Zhang Y, *et al.* (Zhang et al., 2014) showed that freshly isolated astrocytes specifically express higher levels of LAT1 mRNA compared LAT2 mRNA (Zhang et al., 2014). Even though the mRNA expression does not necessarily correspond to protein abundance and dominance (Taslimifar et al., 2017), and although there is no report on the astrocytic transport activity of LAT1 in non-cultured cell assays, we have additionally checked whether considering LAT1 instead of LAT2 as dominant astrocytic AAT would modify our conclusion on the functional polarity of MBECs with either asymmetric bi-directional kinetics and/or asymmetric distribution of

LAT1 in MBECs. To this end, we assumed LAT1 to be the dominant astrocytic LNAA transporter, replacing LAT2 in the astrocyte. In this situation, given that the intra-compartmental fluxes depend on the choice of the dominant transporters, we simply substituted the parameters of Eq. (7) (Methods section) with those of LAT1 for astrocytes (reported in Suppl. Table 3) and similarly calculated the baseline (pre-stimulus) state of the NVU system as reported in Suppl. Table 3. We then calculated the post-stimulus response of LNAA concentrations (Suppl. Fig.1) upon perturbation of plasma L-tyrosine and L-phenylalanine concentrations (Figs. 2A and 2C). All model parameters except for kinetic parameters specific to LAT1 in the astrocyte (Suppl. Table 3) remain the same as in the nominal model (Table 1). The bi-directional kinetic constant for astrocyte LAT1 is considered equal to the corresponding value in the MBEC LAT1 ($\mathrm{RK}_{\mathrm{LAT1}}$). The time evolution of the plasma concentration of L-tyrosine and L-phenylalanine and of competing LNAAs is plotted as percentage of the baseline values in Suppl. Fig. 1. The error bars given for the baseline concentrations were calculated based on sensitivity studies as described in the Sensitivity analysis section. We compared the model predictions for the ISF response to IP L-tyrosine and L-phenylalanine injection with results of *in vivo* measurements (Bongiovanni et al., 2003; Bongiovanni et al., 2010), in the range from 1 (symmetric case) and 1300 (highly asymmetric bi-directional kinetics(Meier et al., 2002)) (Suppl. Fig. 1A,C). We found a close agreement between our model calculations and experimental measurements assuming asymmetric MBEC LAT1 kinetics (best with $\mathrm{RK}_{\mathrm{LAT1}} = 220$ and 45, respectively, for L-tyrosine and L-phenylalanine IP injection cases), while the model failed to reproduce the experimental data when the bi-directional kinetics of LAT1 were assumed to be symmetric in MBECs (Suppl. Fig. 1A, C).

To evaluate the hypothesis of asymmetric distribution of LAT1 at the BBB, we varied the abluminal to luminal expression ratio of LAT1, $\mathrm{RE}_{\mathrm{LAT1}}$, between 0.01 to 10 (representing highly asymmetric abluminal to luminal expression ratio) while we assumed the bi-directional kinetics of LAT1 to be symmetric ($\mathrm{RK}_{\mathrm{LAT1}} = 1$). The numerical results obtained with asymmetric transporter expression agreed well with *in vivo* experimental data, best for an expression kinetic constant of $\mathrm{RE}_{\mathrm{LAT1}}$=0.12. Taken together, our results show that assuming LAT1 rather than LAT2 as the dominant astrocytic AAT does not affect our conclusion on functional polarity of MBECs with either strong asymmetric kinetics of LAT1 and/or its expression at the BBB. Further exploration of the correlation between astrocyte mRNA is required to characterize the function of LAT1 in astrocytes *in vivo*.

Supplementary Table 3: The kinetic parameters when assuming LAT1 as the dominant LNAA transporter in astrocytes, and the calculated baseline concentration of individual LNAAs in the different brain compartments

	L-tyrosine[a]		L-phenylalanine[b]		
**Parameters**	**Value**				**Unit**
**LAT2 (Astrocyte)**					
$K_{m,abs,LAT1,TL}^{ISF(Ast)}$	64 (Smith et al., 1987)		11 (Smith et al., 1987)		µM
$V_{max,LAT1,TL}$	0.132 (Shank and Campbell, 1984)		0.184 (Shank and Campbell, 1984)		µmol/min
$K_{m,abs,LAT1,CL}^{ISF(Ast)}$	37 (Smith et al., 1987)		52.9 (Smith et al., 1987)		µM
$V_{max,LAT1,CL}$	0.184^{c} (Shank and Campbell, 1984)		0.178^{c} (Shank and Campbell, 1984)		µmol/min
**Calculated baseline concentration[d]**					
$[TL]_b^{MBEC}$	281.4 ± 23.9	16.8±1.1	142.9 ± 24.9	99.7 ±14.7	µM
$[CL]_b^{MBEC}$	1770.9 ±150.3	105.8±6.8	1371.7 ± 238.9	957 ±140.7	µM
$[TL]_b^{ISF}$	1.0 ± 0.1	1.1±0.1	0.4 ± 0.03	0.4 ±0.03	µM
$[CL]_b^{ISF}$	6.4 ± 0.8	7.1±0.8	3.8 ±0.3	3.9 ±0.3	µM
$[TL]_b^{Ast}$	63.0± 7.5	62.4±7	39.6 ± 4.7	38.4 ±4.4	µM
$[CL]_b^{Ast}$	395.7 ± 42.3	392.4±44.1	379.5 ±45.3	368.8 ±42.7	µM
$[TL]_b^{Neu}$	74.6 ± 8.8	75.4±8.7	54.4 ± 6.6	57.3 ±6.4	µM
$[CL]_b^{Neu}$	308.8 ± 40.8	342.9±39.2	376.1 ± 44.0	395 ±43	µM
The bi-directional and expression constant of MBEC LAT1					
RK_{LAT1}	220	1	45	1	-
RE_{LAT1}	1	0.12	1	0.12	-

[a]In this column, TL and CL represent L-tyrosine and L-tyrosine competing LNAAs, respectively. [b]In this column, TL and CL represent L-phenylalanine and L-phenylalanine competing LNAAs, respectively. [c]The kinetic parameters for the mixture of L-tyrosine and L-phenylalanine competing LNAAs are calculated based on Eq.10 (Suppl. Table 1) and the reported Vmax values for individual LNAAs are based on the Leucine measurements [d]The initialization of baseline concentrations is described in Suppl. Table 2.

3 Statistical analysis

Supplementary Table 4: P-values of Student's unpaired t-test for differences between calculated and measured concentrations

	Model parameters		Post-stimulus time (min)							
	RK_{LAT1} [-]	RE_{LAT1} [-]	**30**	**60**	**90**	**120**	**150**	**180**	**210**	**240**
**Fig. 2B**	1	1	$<10^{-15}$	$<10^{-15}$	$<10^{-15}$	$<10^{-15}$	$<10^{-15}$	$<10^{-15}$	$<10^{-15}$	$2.38\cdot10^{-8}$
	10	1	$3.79\cdot10^{-7}$	$2.45\cdot10^{-4}$	$7.95\cdot10^{-3}$	0.056	0.057	0.057	0.057	0.011
	160	1	0.387	0.035	0.906	0.906	0.906	0.387	0.387	0.387
	1300	1	$<10^{-15}$	$<10^{-15}$	$<10^{-15}$	$<10^{-15}$	$<10^{-15}$	$<10^{-15}$	$<10^{-15}$	$<10^{-15}$
**Fig. 2C**	1	0.01	$<10^{-15}$	$<10^{-15}$	$<10^{-15}$	$<10^{-15}$	$<10^{-15}$	$<10^{-15}$	$<10^{-15}$	$<10^{-15}$
	1	0.18	$<10^{-15}$	0.235	0.847	0.329	0.149	0.629	0.847	0.149
	1	1	$<10^{-15}$	$<10^{-15}$	$<10^{-15}$	$<10^{-15}$	$<10^{-15}$	$<10^{-15}$	$<10^{-15}$	$2.38\cdot10^{-8}$
	1	10	$<10^{-15}$	$<10^{-15}$	$<10^{-15}$	$<10^{-15}$	$<10^{-15}$	$<10^{-15}$	$5.01\cdot10^{-10}$	$1.90\cdot10^{-5}$
**Fig. 2E**	1	1	$<10^{-15}$	$<10^{-15}$	$<10^{-15}$	$<10^{-15}$	$1.36\cdot10^{-5}$	0.972	$<10^{-15}$	$<10^{-15}$
	10	1	$1.77\cdot10^{-8}$	$9.60\cdot10^{-12}$	$1.56\cdot10^{-11}$	$6.70\cdot10^{-9}$	0.013	0.807	$8.45\cdot10^{-5}$	0.121
	80	1	$8.96\cdot10^{-8}$	0.990	0.749	0.990	0.047	0.990	0.018	0.826
	1300	1	$<10^{-15}$	$<10^{-15}$	$<10^{-15}$	$<10^{-15}$	$<10^{-15}$	$<10^{-15}$	$<10^{-15}$	$<10^{-15}$
**Fig. 2F**	1	0.01	$<10^{-15}$	$<10^{-15}$	$<10^{-15}$	$<10^{-15}$	$<10^{-15}$	$<10^{-15}$	$<10^{-15}$	$<10^{-15}$
	1	0.11	0.001	0.720	0.720	0.720	0.435	0.720	0.231	0.720
	1	1	$<10^{-15}$	$<10^{-15}$	$<10^{-15}$	$<10^{-15}$	$1.36\cdot10^{-5}$	0.972	$<10^{-15}$	$<10^{-15}$
	1	10	$<10^{-15}$	$<10^{-15}$	$<10^{-15}$	$5.15\cdot10^{-12}$	0.024	$<10^{-15}$	$<10^{-15}$	$<10^{-15}$

	System input	Post-stimulus time (min)				
	[BCH] [µM]	**20**	**50**	**80**	**110**	**170**
**Fig. 5A**	10	$<10^{-15}$	$<10^{-15}$	$<10^{-15}$	$<10^{-15}$	$<10^{-15}$
	30	$2.94\cdot10^{-12}$	0.121	0.662	0.023	0.646
	100	$<10^{-15}$	$<10^{-15}$	$<10^{-15}$	$<10^{-15}$	$<10^{-15}$
**Fig.5.B**	10	$<10^{-15}$	$<10^{-15}$	$<10^{-15}$	$<10^{-15}$	$<10^{-15}$
	30	$1.50\cdot10^{-8}$	0.971	0.045	0.971	0.045
	100	$<10^{-15}$	$<10^{-15}$	$<10^{-15}$	$<10^{-15}$	$<10^{-15}$
**Fig.5.C**	5	$<10^{-15}$	$<10^{-15}$	$<10^{-15}$	$<10^{-15}$	$<10^{-15}$
	17	$<10^{-15}$	0.135	0.470	0.151	0.492
	10	$<10^{-15}$	$<10^{-15}$	$<10^{-15}$	$<10^{-15}$	$<10^{-15}$
**Fig.5.D**	5	$<10^{-15}$	$<10^{-15}$	$<10^{-15}$	$<10^{-15}$	$<10^{-15}$
	17	$<10^{-15}$	0.549	0.071	0.571	0.062
	100	$<10^{-15}$	$<10^{-15}$	$<10^{-15}$	$<10^{-15}$	$<10^{-15}$

	Model parameters		Post-stimulus time (min)							
	RK_{LAT1} [-]	RE_{LAT1} [-]	**30**	**60**	**90**	**120**	**150**	**180**	**210**	**240**
**Suppl. Fig.1.A**	1	1	$<10^{-15}$	$<10^{-15}$	$<10^{-15}$	$<10^{-15}$	$<10^{-15}$	$<10^{-15}$	$<10^{-15}$	$<10^{-15}$
	10	1	$3.32\cdot10^{-7}$	$5.64\cdot10^{-5}$	$6.63\cdot10^{-3}$	0.056	0.057	0.057	0.057	0.011
	220	1	0.139	$2.34\cdot10^{-4}$	0.029	0.472	0.737	0.472	0.394	0.246
	1300	1	$<10^{-15}$	$<10^{-15}$	$<10^{-15}$	$<10^{-15}$	$<10^{-15}$	$<10^{-15}$	$<10^{-15}$	$<10^{-15}$
**Suppl. Fig.1.B**	1	0.01	$<10^{-15}$	$<10^{-15}$	$<10^{-15}$	$<10^{-15}$	$<10^{-15}$	$<10^{-15}$	$<10^{-15}$	$<10^{-15}$
	1	0.12	$3.42\cdot10^{-12}$	0.623	0.627	0.623	0.011	0.627	0.503	0.503
	1	1	$<10^{-15}$	$<10^{-15}$	$<10^{-15}$	$<10^{-15}$	$<10^{-15}$	$<10^{-15}$	$<10^{-15}$	$<10^{-15}$
	1	10	$<10^{-15}$	$<10^{-15}$	$<10^{-15}$	$<10^{-15}$	$<10^{-15}$	$<10^{-15}$	$3.31\cdot10^{-11}$	$9.66\cdot10^{-6}$
**Suppl. Fig.1.C**	1	1	$<10^{-15}$	$<10^{-15}$	$<10^{-15}$	$<10^{-15}$	$8.83\cdot10^{-7}$	0.012	0.262	$6.17\cdot10^{-4}$
	10	1	$7.21\cdot10^{-11}$	$2.40\cdot10^{-8}$	$1.71\cdot10^{-8}$	$1.03\cdot10^{-5}$	$1.62\cdot10^{-4}$	$2.90\cdot10^{-4}$	0.018	$1.58\cdot10^{-4}$
	45	1	0.032	$4.35\cdot10^{-9}$	0.223	0.032	0.045	0.013	0.535	0.017
	1300	1	$<10^{-15}$	$<10^{-15}$	$<10^{-15}$	$<10^{-15}$	$<10^{-15}$	$<10^{-15}$	$<10^{-15}$	$<10^{-15}$
**Suppl. Fig.1.D**	1	0.01	$<10^{-15}$	$<10^{-15}$	$<10^{-15}$	$<10^{-15}$	$<10^{-15}$	$<10^{-15}$	$<10^{-15}$	$<10^{-15}$
	1	0.12	0.025	0.011	0.758	0.719	0.758	0.204	0.758	0.088
	1	1	$<10^{-15}$	$<10^{-15}$	$<10^{-15}$	$<10^{-15}$	$8.83\cdot10^{-7}$	0.012	0.262	$6.17\cdot10^{-4}$
	1	10	$<10^{-15}$	$<10^{-15}$	$<10^{-15}$	$7.36\cdot10^{-11}$	0.023	0.030	$9.05\cdot10^{-11}$	$<10^{-15}$

Supplementary Figure 1

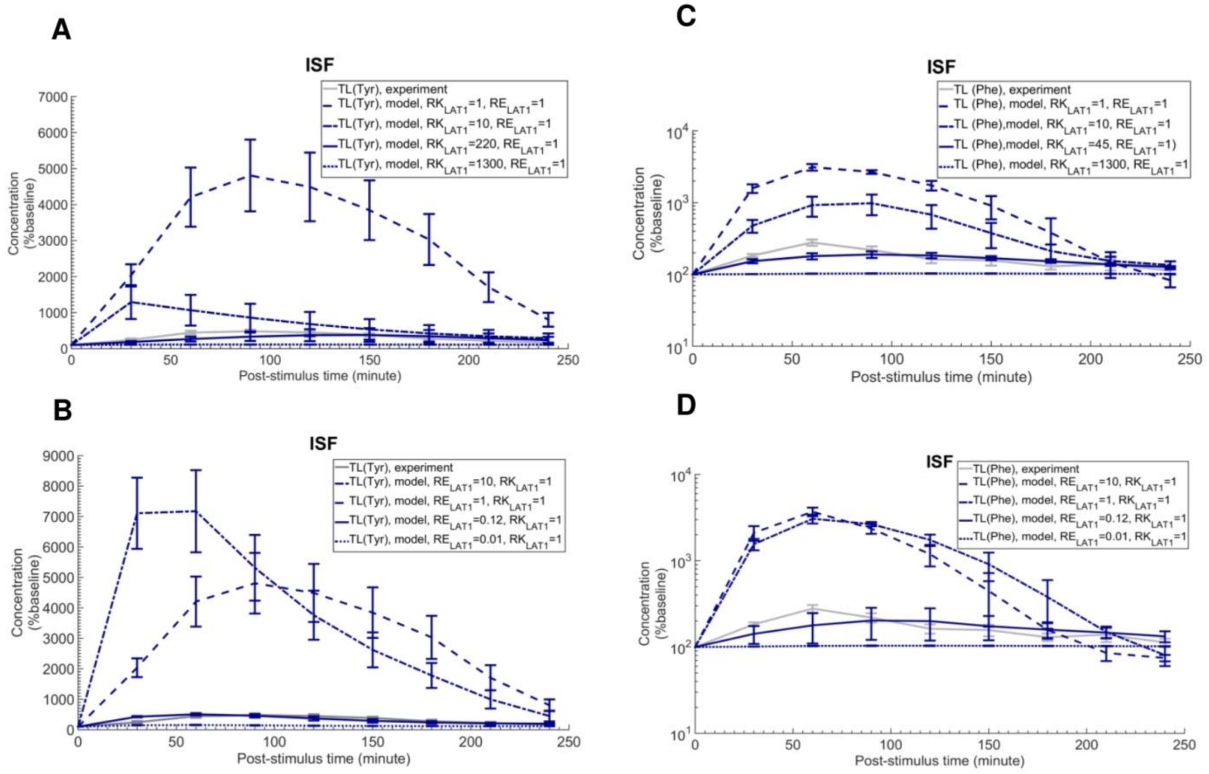

Supplementary Figure 1. The brain ISF concentration response after intraperitoneal injection of L-tyrosine and L-phenylalanine under the assumption of LAT1 as the dominant LNAA transporter in astrocytes. Panels (A) and (B) show the experimental data for L-tyrosine (Tyr) concentration in the brain ISF (prefrontal cortex (PFC)) (Bongiovanni et al., 2003) in response to IP administration of 200 mg/kg L-tyrosine (as plotted in Fig. 2B) compared to the model calculations assuming LAT1 as the dominant transporter in astrocytes (model input as shown in Fig.2A) for various ratios of the bi-directional kinetic constant of LAT1 in MBEC and astrocytes (RK_{LAT1}) as well as different abluminal to luminal expression ratio of LAT1 (RE_{LAT1}). In Panels (A) and (B), the ISF baseline values for L-tyrosine are 1.0 and 1.1 µM (Suppl. Table 3), respectively. Each experimental data point represents the mean ± SD for three (plasma) and four to eight (ISF) animals (Bongiovanni et al., 2003). The error bar associated with model calculations indicate the standard deviation determined based on the sensitivity analysis results. Panel (C) and (D) show the experimental data for L-phenylalanine (Phe) concentration in the brain ISF (prefrontal cortex (PFC)) (Bongiovanni et al., 2010) in response to IP administration of 200 mg/kg L- phenylalanine (as plotted

in Fig. 2D), versus the model calculations (model input is shown in Fig.2D) for various ratios of the bi-directional kinetic constant of LAT1 in MBEC and astrocytes ($\mathrm{RK_{LAT1}}$) as well as different abluminal to luminal expression ratios of LAT1 ($\mathrm{RE_{LAT1}}$). In panels (C) and (D), the ISF baseline value for L-phenylalanine is 0.4 µM as reported in Suppl. Table 3. In Panels (A), (B), (C) and (D), the differences between the output of the symmetric model ($\mathrm{RK_{LAT1}} = 1$ and $\mathrm{RE_{LAT1}} = 1$) and experimental measurements are statistically significant at all post-stimulus time points ($p<0.001$, Suppl. Table 4) with the exception of 180 and 210 min in Panels (C) and (D). In contrast, there is no significant difference between the experimental measurements and the model calculations with $\mathrm{RK_{LAT1}}$ =220 and $\mathrm{RE_{LAT1}} = 1$ (Panel A), $\mathrm{RK_{LAT1}} = 1$ and $\mathrm{RE_{LAT1}} = 0.12$ (Panels (B) and (D)), and $\mathrm{RK_{LAT1}} = 45$ and $\mathrm{RE_{LAT1}} = 1$ (Panel C), with the exception of the 30 min post-stimulus time point in Panel (B) and the 60 min point in Panels (A) and (C) (Suppl. Table 4).

4 References

Amorini, A.M., Lazzarino, G., Di Pietro, V., Signoretti, S., Lazzarino, G., Belli, A., et al. (2017). Severity of experimental traumatic brain injury modulates changes in concentrations of cerebral free amino acids. *Journal of cellular and molecular medicine* 21(3)**,** 530-542.

Anderova, M., Vorisek, I., Pivonkova, H., Benesova, J., Vargova, L., Cicanic, M., et al. (2011). Cell Death/Proliferation and Alterations in Glial Morphology Contribute to Changes in Diffusivity in the Rat Hippocampus after Hypoxia—Ischemia. *Journal of Cerebral Blood Flow & Metabolism* 31(3)**,** 894-907.

Bak, L.K., Johansen, M.L., Schousboe, A., and Waagepetersen, H.S. (2012). Valine but not leucine or isoleucine supports neurotransmitter glutamate synthesis during synaptic activity in cultured cerebellar neurons. *Journal of neuroscience research* 90(9)**,** 1768-1775.

Banay-Schwartz, M., Kenessey, A., DeGuzman, T., Lajtha, A., and Palkovits, M. (1992). Protein content of various regions of rat brain and adult and aging human brain. *Age* 15(2)**,** 51-54.

Bongiovanni, R., Kirkbride, B., Newbould, E., Durkalski, V., and Jaskiw, G.E. (2010). Relationships between large neutral amino acid levels in plasma, cerebrospinal fluid, brain microdialysate and brain tissue in the rat. *Brain research* 1334**,** 45-57.

Bongiovanni, R., Yamamoto, B.K., Simpson, C., and Jaskiw, G.E. (2003). Pharmacokinetics of systemically administered tyrosine: a comparison of serum, brain tissue and in vivo microdialysate levels in the rat. *Journal of neurochemistry* 87(2)**,** 310-317.

Braun, D., Kinne, A., Bräuer, A.U., Sapin, R., Klein, M.O., Köhrle, J., et al. (2011). Developmental and cell type-specific expression of thyroid hormone transporters in the mouse brain and in primary brain cells. *Glia* 59(3)**,** 463-471.

Bröer, A., Tietze, N., Kowalczuk, S., Chubb, S., Munzinger, M., Bak, L.K., et al. (2006). The orphan transporter v7-3 (slc6a15) is a Na+-dependent neutral amino acid transporter (B0AT2). *Biochemical Journal* 393(1)**,** 421-430.

Currie, P.J., Chang, N., Luo, S., and Anderson, G.H. (1995). Microdialysis as a tool to measure dietary and regional effects on the complete profile of extracellular amino acids in the hypothalamus of rats. *Life sciences* 57(21)**,** 1911-1923.

Hosseini-Sharifabad, M., and Nyengaard, J.R. (2007). Design-based estimation of neuronal number and individual neuronal volume in the rat hippocampus. *Journal of neuroscience methods* 162(1)**,** 206-214.

Kandera, J., Levi, G., and Lajtha, A. (1968). Control of cerebral metabolite levels: II. Amino acid uptake and levels in various areas of the rat brain. *Archives of biochemistry and biophysics* 126(1)**,** 249-260.

Kim, D.K., Kim, I.J., Hwang, S., Kook, J.H., Lee, M.C., Shin, B.A., et al. (2004). System L-amino acid transporters are differently expressed in rat astrocyte and C6 glioma cells. *Neuroscience Research* 50(4)**,** 437-446. doi: DOI 10.1016/j.neures.2004.08.003.

Meier, C., Ristic, Z., Klauser, S., and Verrey, F. (2002). Activation of system L heterodimeric amino acid exchangers by intracellular substrates. *EMBO J* 21(4)**,** 580-589.

Rao, K.R., Vemuri, M.C., and Murthy, C.R. (1995). Synaptosomal transport of branched chain amino acids in young, adult and aged rat brain cortex. *Neuroscience letters* 184(2)**,** 137-140.

Ren, J., Aika, Y., Heizmann, C., and Kosaka, T. (1992). Quantitative analysis of neurons and glial cells in the rat somatosensory cortex, with special reference to GABAergic neurons and parvalbumin-containing neurons. *Experimental brain research* 92(1)**,** 1-14.

Segawa, H., Fukasawa, Y., Miyamoto, K., Takeda, E., Endou, H., and Kanai, Y. (1999). Identification and functional characterization of a Na+-independent neutral amino acid transporter with broad substrate selectivity. *Journal of Biological Chemistry* 274(28)**,** 19745-19751. doi: DOI 10.1074/jbc.274.28.19745.

Segel, I.H. (1975). *Biochemical calculations.* Wiley.

Setou, M., Hayasaka, T., and Yao, I. (2004). Axonal transport versus dendritic transport. *Journal of neurobiology* 58(2)**,** 201-206.

Shank, R.P., and Campbell, G.L. (1984). Amino acid uptake, content, and metabolism by neuronal and glial enriched cellular fractions from mouse cerebellum. *The Journal of neuroscience* 4(1)**,** 58-69.

Smith, Q.R., Momma, S., Aoyagi, M., and Rapoport, S.I. (1987). Kinetics of neutral amino acid transport across the blood-brain barrier. *Journal of neurochemistry* 49(5)**,** 1651-1658.

Stewart, C. (1918). Weights of various parts of the brain in normal and underfed albino rats at different ages. *Journal of Comparative Neurology* 29(5)**,** 511-528.

Taslimifar, M., Oparija, L., Verrey, F., Kurtcuoglu, V., Olgac, U., and Makrides, V. (2017). Quantifying the relative contributions of different solute carriers to aggregate substrate transport. *Scientific reports* 7**,** 40628.

Tilgmann, C., Melen, K., Lundström, K., Jalanko, A., Julkunen, I., Kalkkinen, N., et al. (1992). Expression of recombinant soluble and membrane-bound catechol O-methyltransferase in

eukaryotic cells and identification of the respective enzymes in rat brain. *European Journal of Biochemistry* 207(2)**,** 813-821.

Yudkoff, M., Daikhin, Y., Grunstein, L., Nissim, I., Stern, J., Pleasure, D., et al. (1996a). Astrocyte leucine metabolism: Significance of branched-chain amino acid transamination. *Journal of neurochemistry* 66(1)**,** 378-385.

Yudkoff, M., Daikhin, Y., Nelson, D., Nissim, I., and Erecińska, M. (1996b). Neuronal metabolism of branched-chain amino acids: Flux through the aminotransferase pathway in synaptosomes. *Journal of neurochemistry* 66(5)**,** 2136-2145.

Zhang, Y., Chen, K., Sloan, S.A., Bennett, M.L., Scholze, A.R., O'Keeffe, S., et al. (2014). An RNA-sequencing transcriptome and splicing database of glia, neurons, and vascular cells of the cerebral cortex. *Journal of Neuroscience* 34(36)**,** 11929-11947.
